# Endocrine and metabolic determinants of cardiometabolic risk in mild autonomous cortisol secretion

**DOI:** 10.1016/j.ebiom.2026.106126

**Published:** 2026-01-22

**Authors:** Alessandro Prete, Lida Abdi, Marco Canducci, Elina L. van den Brandhof, Ariadna Albors-Zumel, Carl Jenkinson, Lorna C. Gilligan, Yuanqing Zhang, Ludger Visser, Vasileios Chortis, Lukáš Najdekr, Andris Jankevics, Gavin R. Lloyd, Catherine L. Winder, Stylianos Tsagarakis, Katharina Lang, Magdalena Macech, Vanessa Fell, Ivana D. Vodanovic, Giuseppe Reimondo, Ljiljana V. Marina, Timo Deutschbein, Maria Balomenaki, Michael W. O'Reilly, Tomasz Bednarczuk, Tina Dusek, Aristidis Diamantopoulos, Miriam Asia, Agnieszka Kondracka, Kai Yu, Jimmy R. Masjkur, Marcus Quinkler, Grethe Å. Ueland, M. Conall Dennedy, Felix Beuschlein, Antoine Tabarin, Martin Fassnacht, Miomira Ivovic, Massimo Terzolo, Darko Kastelan, William F. Young, Konstantinos N. Manolopoulos, Urszula Ambroziak, Dimitra A. Vassiliadi, Irina Bancos, Alice J. Sitch, Angela E. Taylor, Peter Tino, Michael Biehl, Warwick B. Dunn, Wiebke Arlt

**Affiliations:** a Department of Metabolism and Systems Science, School of Medical Sciences, College of Medicine and Health, University of Birmingham, Birmingham, UK; b National Institute for Health and Care Research (NIHR) Birmingham Biomedical Research Centre, Birmingham, UK; c Centre for Endocrinology, Diabetes and Metabolism, Birmingham Health Partners, Birmingham, UK; d Department of Endocrinology, Queen Elizabeth Hospital, University Hospitals Birmingham NHS Foundation Trust, Birmingham, UK; e Medical Research Council Laboratory of Medical Sciences, London, UK; f School of Computer Science, University of Birmingham, Birmingham, UK; g Bernoulli Institute for Mathematics, Computer Science and Artificial Intelligence, University of Groningen, Groningen, the Netherlands; h Department of Neurology, University Medical Center Groningen, University of Groningen, Groningen, the Netherlands; i Department of Sociology and Social Research, University of Trento, Trento, Italy; j Phenome Centre Birmingham, School of Biosciences, University of Birmingham, Edgbaston, Birmingham, B15 2TT, UK; k Department of Endocrinology and Metabolism, Diabetes Centre, Evangelismos Hospital, Athens, Greece; l Department of Internal Medicine and Endocrinology, Medical University of Warsaw, Warsaw, Poland; m Division of Endocrinology, Metabolism, Diabetes and Nutrition, Department of Internal Medicine, Mayo Clinic, Rochester, MN, USA; n Department of Endocrinology, University Hospital Centre Zagreb, Zagreb, Croatia; o Division of Internal Medicine, University of Turin, San Luigi Hospital, Turin, Italy; p Department for Obesity, Reproductive and Metabolic Disorders, Clinic for Endocrinology, Diabetes and Metabolic Diseases, University Clinical Centre of Serbia, Faculty of Medicine, University of Belgrade, Belgrade, Serbia; q Department of Internal Medicine I, Division of Endocrinology and Diabetes, University Hospital, University of Würzburg, Würzburg, Germany; r Medicover Oldenburg MVZ, Oldenburg, Germany; s Department of Medicine, Royal College of Surgeons in Ireland, University of Medicine and Health Sciences, Dublin, Republic of Ireland; t Department of Medicine III and Institute of Clinical Chemistry and Laboratory Medicine, Technische Universität Dresden, Dresden, Germany; u Endocrinology in Charlottenburg, Berlin, Germany; v Department of Endocrinology, Haukeland University Hospital, Bergen, Norway; w Department of Endocrinology, University Hospital Galway, Newcastle, Galway, Ireland; x Klinik für Endokrinologie, Diabetologie und Klinische Ernährung, Universitäts-Spital Zürich (USZ) und Universität Zürich (UZH), Zurich, Switzerland; y Medizinische Klinik und Poliklinik IV, Ludwig-Maximilians-Universität München, Munich, Germany; z The LOOP Zurich - Medical Research Center, Zurich, Switzerland; aa Service d'Endocrinologie, Centre Hospitalier Universitaire, Hopital du Haut Leveque, Pessac, France; ab European Reference Network on Rare Endocrine Conditions (Endo-ERN), Amsterdam, the Netherlands; ac Department of Applied Health Sciences, School of Health Sciences, College of Medicine and Health, University of Birmingham, Birmingham, UK; ad Department of Biochemistry, Cell and Systems Biology, Centre for Metabolomics Research, Institute of Systems, Molecular, and Integrative Biology, University of Liverpool, Liverpool, UK; ae Institute of Clinical Sciences, Imperial College London, London, UK

**Keywords:** Adrenal incidentaloma, Cortisol excess, Mild autonomous cortisol secretion, Non-functioning adrenal tumour, Cushing's syndrome

## Abstract

**Background:**

Benign adrenal tumours, found in 1–7% of adults, can be non-functioning (NFAT) or show mild autonomous cortisol secretion (MACS), i.e., biochemical cortisol excess without manifestations of Cushing's syndrome (CS). MACS occurs in 20–50% of cases and is linked to increased cardiometabolic burden.

**Methods:**

In a cross-sectional study, we analysed the 24-h urinary steroid metabolome of 1305 prospectively recruited patients (649 NFAT, 591 MACS, 65 adrenal CS) by tandem mass spectrometry. A sub-group (104 NFAT, 140 MACS, 47 adrenal CS) underwent untargeted serum metabolome analysis by mass spectrometry. Data were analysed using linear regression and supervised machine learning.

**Findings:**

Alongside the expected increase in glucocorticoid excretion from NFAT over MACS to adrenal CS, steroid analysis revealed decreased classic androgen metabolite excretion. By contrast, adrenal-derived 11-oxygenated androgen metabolites remained unchanged. Both glucocorticoid metabolites and the major 11-oxygenated androgen metabolite 11β-hydroxyandrosterone correlated with a higher risk of hypertension and type 2 diabetes. Untargeted metabolome analysis revealed gradual changes towards a lipotoxic phenotype from NFAT over MACS to adrenal CS, with perturbations in glycerophospholipids, lysoglycerophospholipids, triacylglycerides, ceramides, sphingolipids, and acylcarnitines.

**Interpretation:**

MACS represents a metabolic continuum between NFAT and adrenal CS. Increased activity of the adrenal enzyme 11β-hydroxylase (CYP11B1), which catalyses key steps in cortisol and 11-oxygenated androgen biosynthesis, may contribute to steroid excess and cardiometabolic morbidity in MACS. These findings suggest that CYP11B1 may be a potential therapeutic target to ameliorate metabolic dysfunction in MACS.

**Funding:**

10.13039/501100018952NIHR Birmingham Biomedical Research Centre; Diabetes UK; 10.13039/100010269Wellcome Trust; 10.13039/501100000780European Commission; 10.13039/501100000265Medical Research Council.

**Research in context**

**Evidence before this study**

We searched PubMed and Web of Science from inception to July 2025 for studies assessing the metabolic profile of patients with benign adrenal tumours and mild autonomous cortisol secretion (MACS). Search terms included combinations of “adrenal tumour”, “adrenal mass”, “adrenal adenoma”, “adrenal incidentaloma”, “mild autonomous cortisol secretion”, “subclinical Cushing's syndrome”, “Cushing's syndrome”, “metabolomic”, “metabolome”, “steroid profiling”, and “lipidome”. We included studies in any language that investigated cortisol-related metabolic alterations in MACS using mass spectrometry-based techniques in serum or urine. Four urinary multi-steroid profiling studies (196 patients with MACS in total) reported increased glucocorticoid excretion, reduced classic androgen metabolite excretion, and an elevated glucocorticoid-to-androgen ratio in MACS compared to non-functioning adrenal tumours or controls. In 72 patients with MACS, a lower day/night ratio of urinary steroid excretion suggested altered circadian steroid regulation. One targeted plasma metabolomics study comparing 31 patients with MACS to non-functioning tumours, overt Cushing's syndrome, and controls reported changes in carnitine, amino acids, biogenic amines, and phosphatidylcholines.

**Added value of this study**

This study integrates urinary multi-steroid profiling and untargeted serum metabolomics with clinical outcomes in the largest prospective MACS cohort to date. Using high-resolution mass spectrometry and supervised machine learning, we identified metabolic signatures in both urine and serum that reflect disrupted steroidogenesis and systemic metabolic alterations in MACS. Notably, we show that increased urinary excretion of glucocorticoid and 11-oxygenated androgen metabolites is associated with a higher risk of hypertension and type 2 diabetes. These findings support a mechanistic role of the enzyme 11β-hydroxylase (CYP11B1), which catalyses key steps in cortisol and 11-oxygenated androgen biosynthesis.

**Implications of all the available evidence**

Our findings support the use of steroid and untargeted metabolomics to improve diagnostic accuracy and risk stratification in MACS. The observed metabolic patterns—particularly those linked to CYP11B1—highlight this enzyme as a potential central driver of cortisol-related metabolic risk and therapeutic target. These insights provide a biological rationale for future trials of CYP11B1 inhibitors in MACS and suggest that metabolomic profiling could guide more personalised and mechanistic treatment approaches for patients with adrenal incidentalomas.

## Introduction

Adrenal tumours are estimated to affect 1–7% of adults and are mostly detected incidentally during cross-sectional imaging arranged for reasons other than adrenal disease.^1^^,^^2^ About 90% of adrenal incidentalomas are benign and can be non-functioning (NFAT; 40–70% of cases) or cause adrenal hormone excess (30–60% of cases).^2^ Mild autonomous cortisol secretion (MACS) is the most common hormonal abnormality, defined as the failure to suppress serum cortisol after 1 mg of dexamethasone (1 mg dexamethasone suppression test, 1 mg-DST) in the absence of typical signs of overt cortisol excess, i.e., Cushing's syndrome (CS). MACS is diagnosed in 20–50% of patients with benign adrenal incidentalomas.^1^^,^^2^ MACS predominantly affects postmenopausal women and warrants regular assessment for hypertension and type 2 diabetes.^3^ Furthermore, several studies have linked MACS to an increased risk of cardiometabolic disease, frailty, and mortality.^1^, ^2^, ^3^, ^4^, ^5^ As a result, the 2023 International Guideline on the management of adrenal incidentalomas recommended that virtually all cases should be screened for MACS.^2^

**Fig. 1:**
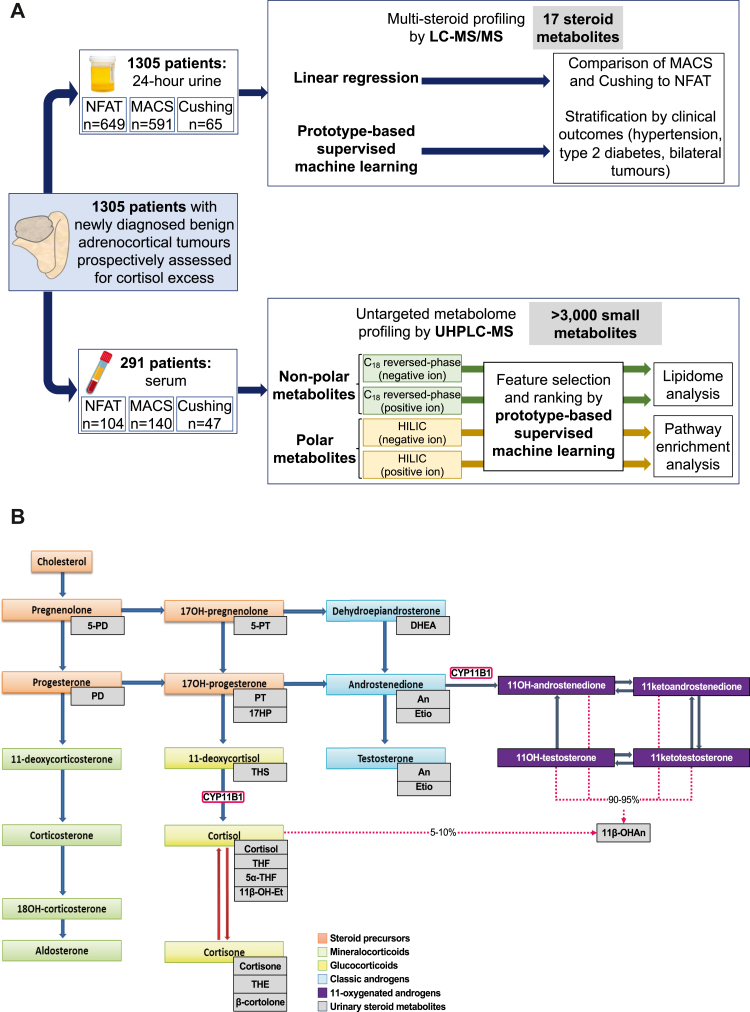
**Study outline and adrenal steroidogenesis overview**. Panel A: Study outline. Panel B: Schematic overview of adrenal steroidogenesis and corresponding urinary steroid metabolites. The key role of 11β-hydroxylase (CYP11B1) in glucocorticoid and 11-oxygenated androgen generation is highlighted. In healthy subjects, 90–95% and 5–10% of 11β-hydroxyandrosterone (11β-OHAn) is generated through the downstream metabolism of 11-oxygenated androgens and cortisol, respectively. Abbreviations: LC-MS/MS, liquid chromatography-tandem mass spectrometry; MACS, mild autonomous cortisol secretion; NFAT, non-functioning adrenal tumour; UHPLC-MS, ultra-high performance liquid chromatography-mass spectrometry. For steroid abbreviations, see Supplemental Table S1.

Considering the high prevalence of adrenal tumours and that as many as half of the cases can have abnormal 1 mg-DST results, MACS is potentially a vastly underestimated contributor to cardiometabolic health in the general population. However, very little is known about its biological underpinnings and biomarkers to identify patients with clinically relevant cortisol excess and guide management are lacking.^2^ In this study, we used mass spectrometry approaches for the analysis of the steroid metabolome and global untargeted metabolome in a large prospectively collected cohort of patients with benign adrenocortical tumours and varying degrees of cortisol excess (NFAT, MACS, adrenal CS), followed by data analysis using supervised machine learning and linear regression (Fig. 1A). This was done to delineate mechanistic insights and to identify potential biomarkers associated with MACS and its associated cardiometabolic disease burden, namely type 2 diabetes and hypertension.

## Methods

### Study design

We analysed prospectively collected serum and urine samples from the EURINE-ACT study, a cross-sectional, prospective, multicentre study assessing the 24-h urinary steroid metabolome and clinical characteristics of adults with newly diagnosed adrenal tumours.^3^^,^^6^ We included 1305 EURINE-ACT study participants (aged ≥18 years) with benign adrenocortical adenomas ≥1 cm and available 1 mg-DST, recruited from 2011 to 2016 through 14 participating adrenal tumour specialist centres in 11 countries. MACS was defined as failure to suppress morning serum cortisol to 50 nmol/L or less after administration of 1 mg of dexamethasone orally at 11:00 p.m. the preceding night (1 mg-DST) in the absence of characteristic CS features, i.e., proximal myopathy, moon face, dorsocervical and supraclavicular fat pads, purple striae.^2^ If typical CS features were present in participants failing to suppress in the 1 mg-DST, they were defined as having CS. Participants suppressing morning cortisol in the 1 mg-DST to 50 nmol/L or less were categorised as having a non-functioning adrenal tumour (NFAT). Patients with MACS were analysed as a whole and were also subdivided according to serum cortisol levels after the 1 mg-DST, to reflect increasing degrees of cortisol excess: 51–138 nmol/L (MACS-1); >138 nmol/L (MACS-2).^7^ Adrenocorticotropic hormone (ACTH)-dependent cortisol excess in MACS and CS was ruled out by measurement of ACTH and/or serum dehydroepiandrosterone sulfate (DHEAS).

All 1305 patients provided a 24-h urine collection at the time of the baseline assessment for their newly diagnosed adrenal tumours (Table 1). A total of 772 EURINE-ACT patients had provided a serum sample; of these, 291 were selected for untargeted serum metabolome profiling (Table 1). Subjects with NFAT, MACS, and CS, respectively, were matched for BMI and prevalence of hypertension, type 2 diabetes, and dyslipidaemia to minimise the confounding effect of body weight and comorbidities on the untargeted metabolome. Criteria to define comorbidities are described in.^3^ Information on the smoking status of EURINE-ACT patients was not available.

**Table 1:** Clinical characteristics of the patients who underwent urinary steroid metabolome and serum untargeted metabolome profiling.

	NFAT (n = 649)	MACS-1 (n = 451)	MACS-2 (n = 140)	Adrenal CS (n = 65)	p-value
**Total cohort used for multi-steroid profiling**					
Women, n (%)	416 (64.1)	303 (67.2)	103 (73.6)	56 (86.2)	0.001
Age at diagnosis (years)	58 (51–65)	64 (56–71)	63 (54–69)	48 (38–60)	<0.001
BMI (kg/m^2^)	29.4 (25.8–33.9)	28.8 (25.1–33.1)	28.6 (24.0–32.9)	28.7 (25.2–31.7)	<0.001
Hypertension, n (%)	416 (64.1)	339 (75.2)	107 (76.4)	47 (72.3)	<0.001
Type 2 diabetes, n (%)	171 (26.4)	145 (32.2)	47 (33.7)	20 (31.5)	0.122
Bilateral tumours, n (%)	107 (16.5)	136 (30.2)	42 (30.0)	13 (20.0)	<0.001

	NFAT (n = 104)	MACS-1 (n = 70)	MACS-2 (n = 70)	Adrenal CS (n = 47)	p-value
**Sub-cohort used for untargeted serum metabolome analysis**					
Women, n (%)	83 (79.8)	70 (100)	70 (100)	42 (89.4)	<0.001
Age at diagnosis (years)	58 (48–66)	64 (56–68)	64 (55–69)	48 (41–60)	<0.001
BMI (kg/m^2^)	28.8 (26.0–32.8)	29.1 (25.2–33.7)	29.3 (24.5–34.9)	28.9 (25.2–32.9)	0.941
Hypertension, n (%)	72 (69.2)	51 (72.9)	56 (80.0)	33 (70.2)	0.447
Type 2 diabetes, n (%)	30 (28.8)	25 (35.7)	25 (35.7)	14 (29.8)	0.694
Dyslipidaemia, n (%)	30 (28.8)	26 (37.1)	29 (41.4)	10 (21.3)	0.088

Clinical information was obtained at the time of adrenal tumour diagnosis. Values are reported as median (interquartile range), unless otherwise stated. The trend for differences across the groups was assessed using one-way ANOVA (for numerical variables) and chi-square test (for categorical variables). Abbreviations: BMI, body mass index; CS, Cushing's syndrome; MACS-1, mild autonomous cortisol secretion (1 mg-overnight dexamethasone test cortisol 51–138 nmol/L); MACS-2, mild autonomous cortisol secretion (1 mg-overnight dexamethasone suppression test cortisol >138 nmol/L); NFAT, non-functioning adrenal tumour (1 mg-overnight dexamethasone suppression test cortisol ≤50 nmol/L).

### Urinary multi-steroid profiling analysis

From each included 24-h urine collection, stored at −20 °C before analysis, 400 μL aliquots were used for steroid extraction before multi-steroid profiling with identification and quantification of 17 distinct urinary steroid metabolites (Fig. 1B) by a validated LC-MS/MS method previously described in^6^ (Steroid Metabolome Analysis Core, University of Birmingham, UK). In brief, after the addition of deuterated steroid standards (DHEA-d6, Cortisol-d4, Etio-d5, THE-d5, THS-d5), samples were deconjugated by hydrolysis. Thereafter, the solution underwent solid phase extraction using Sep Pak C18 cartridges before mass spectrometry analysis. A Waters Xevo mass spectrometer with an ACQUITY ultra-high performance chromatography system with an HSS T3, 1.8 μm, 1.2 × 50 mm column was used to analyse the steroids in positive ionisation mode. The steroid panel did not include any mineralocorticoid metabolites.

### Untargeted serum metabolome analysis

All serum samples were stored at −80 °C before analysis; 50 μL aliquots that had not previously undergone a freeze–thaw cycle were used for untargeted metabolome analysis (Phenome Centre, University of Birmingham, UK). Two assays were applied to increase the coverage of metabolite features detected; polar (water-soluble) metabolites were analysed by hydrophilic interaction chromatography (HILIC) UHPLC-MS, and non-polar (lipid) metabolites by a C18 reversed-phase lipidomics UHPLC-MS. All samples were analysed separately in positive and negative ion modes to increase the number of metabolites detected. Raw data files were deconvoluted using the XCMS software. Intensity drift was corrected for each metabolite feature using the Quality Control-Robust Spline Correction algorithm. Metabolite features defined as of low quality were removed from the dataset before data analysis (metabolite features with QC RSD >30% and detected in less than 90% of QC samples). Metabolite features were annotated using the in-house BEAMS software using MS1 data, an in-house retention time library using retention times and the mass spectral library mzCloud using MS/MS data. Metabolites were reported to MSI levels 1–3 depending on the data used for annotation.^8^ All non-lipid metabolites were grouped into classes based on KEGG metabolic pathway involvement by applying pathway enrichment analysis in MetaboAnalyst. All lipids were grouped based on lipid class. A more detailed description of the method can be found in the Supplemental Material.

### Overview of employed supervised machine learning classifiers

Generalised Matrix Learning Vector Quantisation (GMLVQ) and Ordinal Regression (OR) classifiers were used to dissect the metabolome data to create a representative example, i.e., prototype, for each class (e.g., NFAT, MACS, CS). The machine learning algorithm then compares the metabolome profile of each patient to the prototype of interest and determines how “close” the sample is to that prototype. In essence, these methods are trained to a) construct the class prototypes, b) compare samples to the prototypes based on the learnt distance measure, c) determine how accurately they can separate samples belonging to different classes, and, crucially, d) establish in a multi-variate framework how relevant each metabolite feature is in this separation. The main difference between the two methods is that OR assumes that there is a natural order of the classes (e.g., NFAT → MACS-1 → MACS-2 → CS), whilst GMLVQ does not. Hence, if the OR algorithm assigns a sample to the wrong class, the error is considered more serious when the two classes are far apart (e.g., mislabelling an NFAT case as CS is more serious than mislabelling an NFAT case as MACS-1). In contrast, GMLVQ assigns the same weight to any mislabelling error. A more detailed description of the methods used can be found in the Supplemental Material.

### Supervised machine learning analysis of multi-steroid profiling data

OR was used for the multi-class classification of NFAT, MACS, and CS, and GMLVQ for two-class problems, since GMLVQ and OR are identical in the two-class context. We also devised a double GMLVQ subspace learning method to gain insights into steroid metabolome changes associated with important clinical characteristics (hypertension, type 2 diabetes, presence of bilateral adrenal tumours) in patients with NFAT and MACS.^9^ A more detailed description is provided in the Supplemental Material.

### Linear regression analysis of multi-steroid profiling data

Associations between 24-h urinary steroid excretion measurements and the variable of interest (categories based on 1 mg-DST results [NFAT, MACS-1, MACS-2, CS]; diagnosis of hypertension; diagnosis of type 2 diabetes; presence of bilateral tumours) were determined by linear regression after log-transformation of all outcomes to reduce the impact of outliers. Separate multiple linear regression models were used for each comparison, using the concentration of each steroid as the dependent variable and age, sex, and BMI and the binary comparison of interest (e.g., hypertension vs. no hypertension) as the independent variables. We confirmed the linear model assumption by visually inspecting the residual plots of the fitted models. Associations between the log-transformed outcome and the variable of interest were expressed as symmetric percentages (sympercents), giving percentage changes of urinary steroid metabolite excretion that are the same in both directions and directly comparable.^10^ Linear regression models were generated using Stata Statistical Software: Release 16 (College Station, TX: StataCorp LLC) and GraphPad Prism 9 (San Diego, CA: GraphPad Software Inc.).

### Supervised machine learning analysis of untargeted serum metabolome data

Relative abundances of the metabolite features were normalised and log-transformed to reduce the impact of outliers, and missing data were imputed by probabilistic principal component analysis (probabilistic PCA, PPCA). PPCA provides a linear generative probabilistic model of the data, trained consistently by treating the missing data as latent variables. Multiple imputed data sets were then generated from the PPCA model, where for each data item with missing observations, the missing values were imputed by sampling from the posterior distribution provided by the PPCA, given the observed features for that data item. GMLVQ and OR classifiers were then applied separately to each imputed dataset to rank polar and non-polar metabolite features according to their learnt relevances. Those featuring consistently within the top 500, across the different imputed data sets and in agreement between the two different methodologies, were selected as informative for biological interpretation (Supplemental Figure S15). This cut-off was chosen to cast the net wide enough to retain sufficient information and capture the most relevant metabolic perturbations whilst limiting confounding noise linked to the untargeted nature of the method and the high number of metabolite features detected. Lipidome and pathway enrichment analyses were separately carried out on the top 500 non-polar and polar metabolite features, respectively. A more detailed description of the methods is provided in the Supplemental Material.

### Multi-steroid profiling and untargeted serum metabolome data correlation

Correlations between absolute urinary steroid concentrations and normalised relative abundances of serum metabolite features were assessed using Spearman rank analysis, focussing on the top 500 serum metabolite features and the 5 steroid metabolites most discriminatory across the NFAT-MACS-CS spectrum. A more detailed description of the methods is provided in the Supplemental Material.

### Study approval

The EURINE-ACT study was registered and approved by the European Network for the Study of Adrenal Tumours (ENSAT), in addition to local institutional review board approvals (University of Birmingham approval number HBRC 11-066; 25/NW/0013). All patient recruitment centres had ethical approval for pseudonymized phenotype recording in the online ENSAT database, and all participants of the EURINE-ACT study provided written informed consent before study participation.

### Role of funders

The funders of the study had no role in the study design, data collection, data analyses, interpretation, writing of report, or decision to submit it for publication. No authors were paid to write this article by a pharmaceutical company or other agency. No authors were precluded from accessing data in the study, and they accept responsibility to submit for publication.

## Results

### Patient characteristics

A total of 1305 prospectively recruited adults with benign adrenal tumours were included (NFAT n = 649 [49.7%], MACS n = 591 [45.3%], CS n = 65 [5.0%]) (Table 1). Patients with MACS were analysed both as a whole group and stratified by their cortisol response to 1 mg-DST (MACS-1 n = 451, MACS-2, n = 140), previously shown to be reflective of increasing degrees of cortisol excess and cardiometabolic burden.^3^ Women constituted 67.3% of the overall cohort and were more likely to be diagnosed with MACS and CS than men. Patients with MACS were significantly older, whilst those with CS were younger than NFAT cases. Prevalence of hypertension and type 2 diabetes was higher in the presence of glucocorticoid excess (hypertension: NFAT 64.1%, MACS 75.4%, CS 72.3%; type 2 diabetes: NFAT 26.4%, MACS 32.5%, CS 31.5%). Bilateral adrenal masses had a twofold higher prevalence in MACS (30.1%) than NFAT (16.5%).

### The MACS steroid metabolome shows distinct changes beyond cortisol excess

**Fig. 2:**
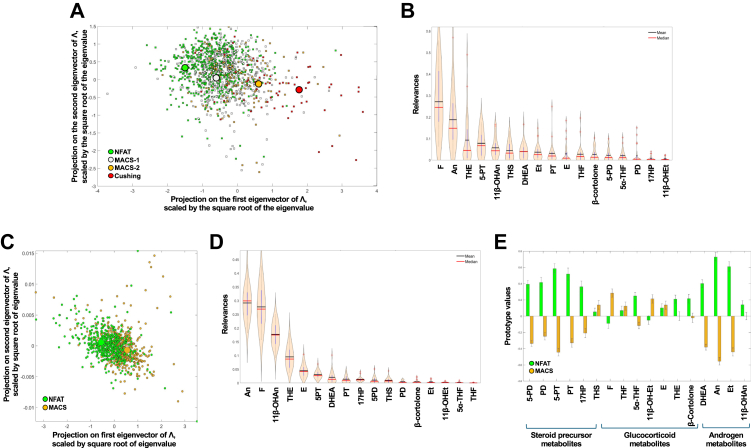
**Supervised machine learning analysis of urinary steroid excretion across benign adrenocortical tumours with different degrees of autonomous cortisol secretion**. Panel A: Two-dimensional ordinal regression embedding of NFAT (n = 649), MACS-1 (n = 451), MACS-2 (n = 140), and adrenal Cushing's syndrome (n = 65) classification; each dot is the projection of the entire steroid metabolome of a patient with adrenal tumours; the big circles represent the class prototypes. Panel B: Violin plots of the steroid metabolite relevances for the multi-class classification. Panel C: Two-dimensional generalised matrix learning vector quantisation embedding of NFAT (n = 649) and MACS (n = 591) classification. Panel D: Violin plots of the steroid metabolite relevances for the NFAT/MACS classification. Panel E: Prototype vectors for the 17 steroid metabolites of NFAT and MACS; columns above and below the 0 represent increased or decreased steroid excretion relative to the other class, respectively, and the height of the column indicates the mean degree of the change across 30 supervised machine learning runs; whiskers represent the standard deviation. Abbreviations: MACS, mild autonomous cortisol secretion; NFAT, non-functioning adrenal tumour. For steroid abbreviations, see Supplemental Table S1.

All 1305 patients underwent 24-h urinary multi-steroid profiling by liquid chromatography-tandem mass spectrometry (LC-MS/MS); a detailed results report is provided in Supplemental Table S1. In a four-class classification task, supervised machine learning showed a gradual progression of cortisol excess from NFAT to MACS-1 to MACS-2 to CS (Fig. 2A), with considerable overlap between classes reflected by a low correct classification rate of 38.7% by OR. Cortisol, its metabolite tetrahydrocortisone, and the classic androgen metabolite androsterone were the most relevant steroids for this classification (Fig. 2B). In a two-class classification task, GMLVQ achieved moderate accuracy in distinguishing NFAT from MACS (Fig. 2C), with an area under the receiver operating characteristic of 0.75 (95% confidence interval 0.74–0.77). The most discriminatory steroids were cortisol, androsterone and 11β-hydroxyandrosterone, the latter a major metabolite of the adrenal-derived 11-oxygenated androgens (Fig. 2D). The computed MACS prototype was characterised by lower androgen metabolite but increased glucocorticoid metabolite excretion (Fig. 2E).

These supervised machine learning results are in keeping with our previously published linear regression data,^3^ showing a progressive increase in excretion of glucocorticoid metabolites and the glucocorticoid precursor metabolite tetrahydro-11-deoxycortisol (THS), and a gradual decrease in classic androgen metabolite excretion across the NFAT-MACS-CS spectrum (Supplemental Figure S1, Supplemental Table S1). By contrast, the 11-oxygenated adrenal androgen metabolite 11β-hydroxyandrosterone did not decrease with increasing cortisol excess (Supplemental Figure S1, Supplemental Table S1). Sub-group analysis by sex revealed that women—regardless of the degree of cortisol excess—had overall lower excretion of most glucocorticoid, androgen, and steroid precursor metabolites than men (Supplemental Figure S2A). Steroid excretion also tended to be lower in patients older than 60 years, with changes more pronounced in CS (Supplemental Figure S2B).

### The MACS steroid metabolome differs according to cardiometabolic disease burden

**Fig. 3:**
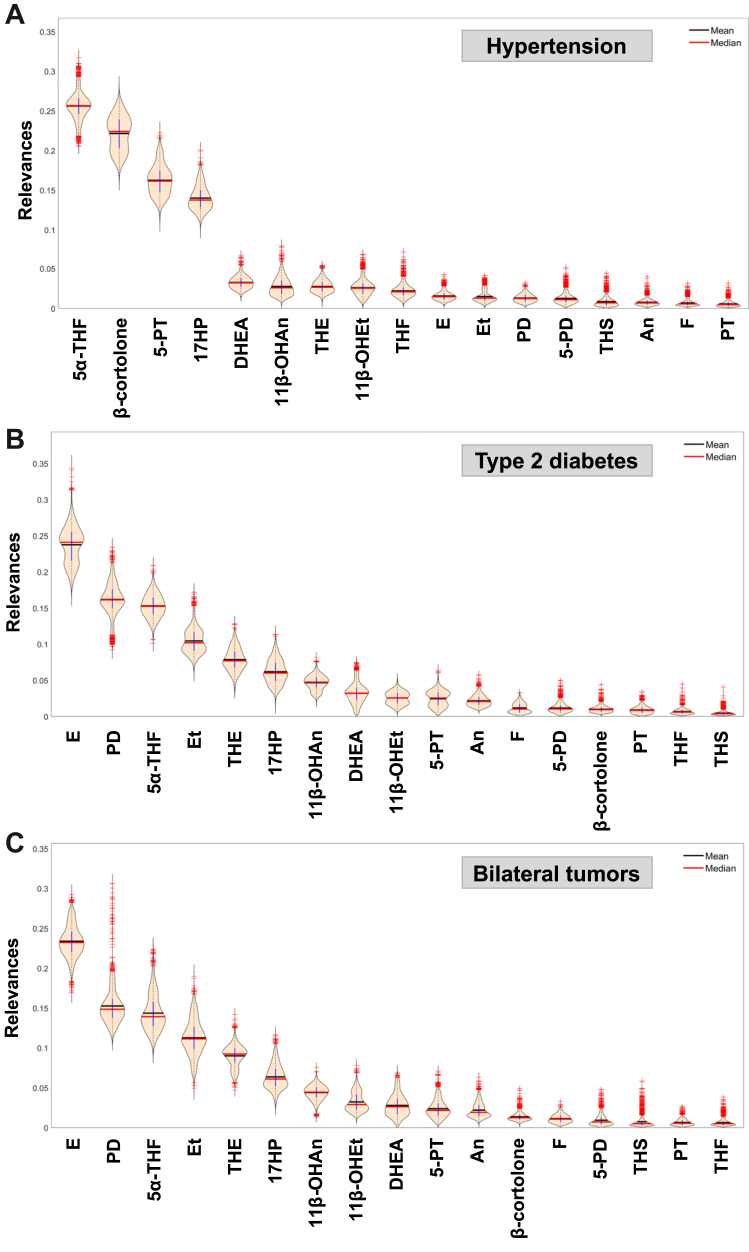
**Discriminative subspace emersion with generalised matrix learning vector quantisation (double GMLVQ) of steroid excretion data analysed for three clinical characteristics**. Violin plots of the steroid metabolite relevances for determining which metabolites are the most discriminatory in MACS with hypertension (n = 446) vs. NFAT with hypertension (n = 416; panel A), MACS with type 2 diabetes (n = 192) vs. NFAT with type 2 diabetes (n = 171; panel B), or MACS with bilateral tumours (n = 178) vs. NFAT with bilateral tumours (n = 107; panel C). Abbreviations: MACS, mild autonomous cortisol secretion; NFAT, non-functioning adrenal tumour. For steroid abbreviations, see Supplemental Table S1.

Both patients with NFAT and MACS who had hypertension tended to have higher urinary glucocorticoid metabolite excretion than those without hypertension, a finding more pronounced for MACS (Supplemental Figure S3). Interestingly, there was higher androgen metabolite excretion in patients with hypertension, most pronounced for 11β-hydroxyandrosterone (Supplemental Figure S3). Double GMLVQ subspace learning identified 5α-tetrahydrocortisol (5α-THF), β-cortolone, and the 17-hydroxypregnenolone metabolite pregnenetriol (5-PT) as the markers most informative for differentiating patients with or without hypertension across MACS and NFAT (Fig. 3A). Because the supervised models quantify multivariate discriminative information rather than univariate effect size, metabolites with high relevance may not necessarily show large differences in mean excretion, particularly when metabolites are correlated within steroidogenic pathways. For example, a metabolite may carry high relevance if it covaries with other pathway-related steroids, even when its mean excretion differs only modestly between groups.

Similar to the findings in patients with hypertension, urinary 11β-hydroxyandrosterone was increased in patients with type 2 diabetes; by contrast, glucocorticoid metabolite excretion was not significantly higher (Supplemental Figure S4). The glucocorticoid metabolites cortisone and 5α-THF, and the progesterone metabolite pregnanediol (PD) were most informative in differentiating NFAT and MACS patients with type 2 diabetes from those without such a diagnosis (Fig. 3B).

### Bilateral adrenal tumours exhibit higher glucocorticoid excess

In patients with bilateral adrenal tumours, both the excretion of glucocorticoid metabolites and THS, the metabolite of the immediate cortisol precursor 11-deoxycortisol, were higher than those with unilateral adrenal tumours, particularly in MACS (Supplemental Figure S5). Glucocorticoid metabolites cortisone and 5α-THF, as well as the progesterone metabolite PD, were most informative for differentiating patients with or without bilateral adrenal tumours across MACS and NFAT (Fig. 3C).

### MACS is associated with adverse changes in the lipidome

A subgroup of 291 patients underwent untargeted serum metabolome profiling by ultra-high performance liquid chromatography-mass spectrometry (UHPLC-MS). Patients with NFAT, MACS, and CS were predominantly women (80–100%) and were matched for body mass index (BMI) and prevalence of hypertension, type 2 diabetes, and dyslipidaemia (Table 1).

**Fig. 4:**
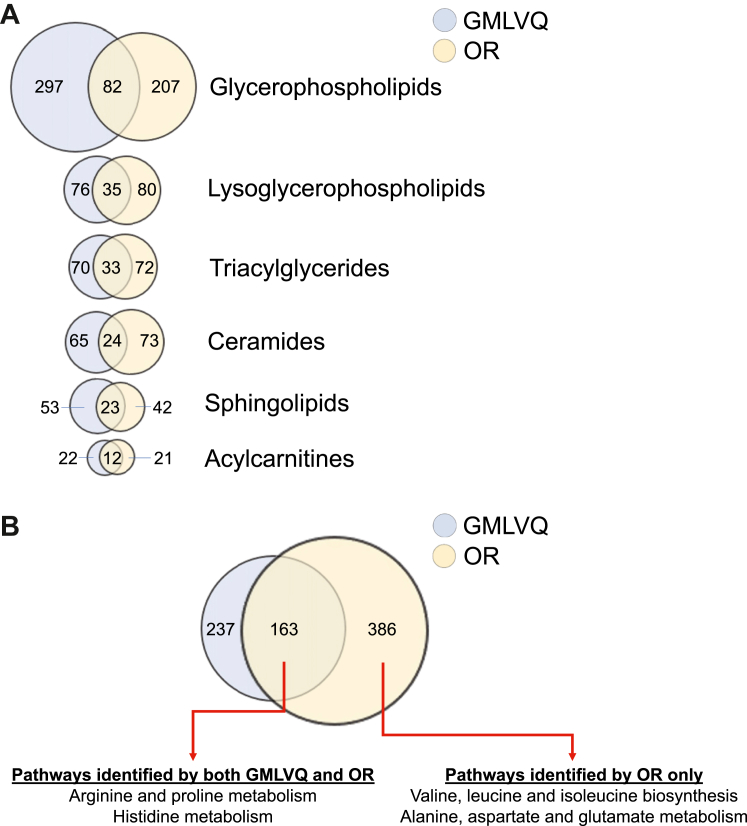
**Main metabolic perturbations observed in patients with benign adrenal tumours**. The number of metabolite features identified by generalised matrix learning vector quantisation (GMLVQ) and ordinal regression (OR) is reported as Venn diagrams. The circle areas and the overlapping areas (showing the number of features identified by both classifiers) are directly proportional to the number of features. Panel A: Lipidome analysis; only lipid classes for which 20 or more metabolite features were selected by GMLVQ or QR are presented. Panel B: Pathway enrichment analysis; the metabolic pathways identified as significant by one or both machine learning classifiers are shown. There were no unique pathways observed in GMLVQ only.

**Fig. 5:**
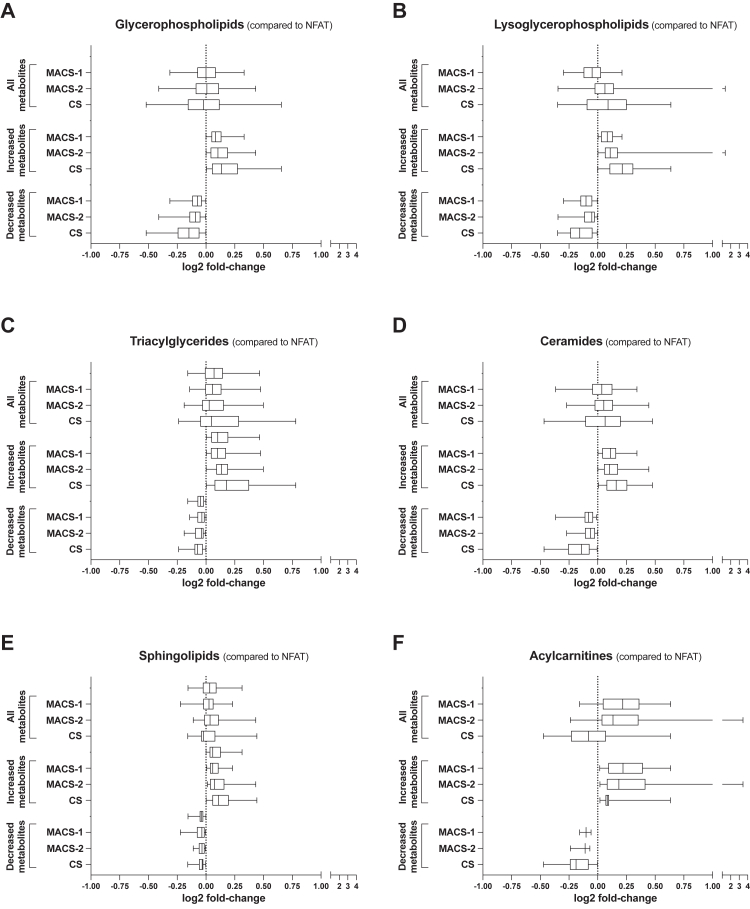
**Lipidome perturbations observed by ordinal regression in patients with benign adrenal tumours**. Relative abundances of metabolite features belonging to the six lipid classes identified by ordinal regression: glycerophospholipids (A); lysoglycerophospholipids (B); triacylglycerides (C); ceramides (D); sphingolipids (E); acylcarnitines (F) (Fig. 4). Results are shown separately for MACS-1 (1 mg-DST cortisol 51–138 nmol/L; n = 70), MACS-2 (1 mg-DST cortisol >138 nmol/L; n = 70), and adrenal CS (n = 47) as fold-changes compared to NFAT (n = 104). Fold-changes are displayed on a log2 scale so that 0 equals no change, and values above and below 0 represent symmetrical degrees of change. Results are shown as boxplots, with boxes representing the median and interquartile range, and whiskers representing the 5th–95th centile. Results are shown separately for all features combined, increased features only, and decreased features only. Abbreviations: 1 mg-DST, 1 mg-overnight dexamethasone suppression test; CS, Cushing's syndrome; MACS, mild autonomous cortisol secretion; NFAT, non-functioning adrenal tumour.

Both GMLVQ and OR classifiers identified the same top six lipid classes as the most perturbed with increasing glucocorticoid excess (Fig. 4A; Supplemental Table S2). Similarly, the degree of perturbation observed across the lipid classes was very similar when assessed by OR (Fig. 5) and GMLVQ (Supplemental Figure S6). Examples of metabolites with the highest statistical significance across NFAT-MACS-CS are presented in Supplemental Figure S7.

Glycerophospholipids were the class with the most metabolite features identified by machine learning, followed by other lipid classes, including triacylglycerides and acylcarnitines. Glycerophospholipids were increasingly perturbed with increasing cortisol excess; this trend was mostly driven by the glycerophospholipid subclasses phosphatidylcholines and—to a lesser extent—phosphatidylethanolamines (Supplemental Figures S8 and S9). Lysoglycerophospholipids were progressively perturbed in MACS-1, MACS-2 and CS compared to NFAT, whilst triacylglycerides and ceramides were more abnormal in CS than in MACS. Obvious trends for sphingolipids were not observed, whilst acylcarnitines—mostly long-chain and medium-chain—were upregulated in MACS and downregulated in CS (Fig. 5, Supplemental Figures S6, S10).

### MACS is associated with changes in arginine & proline and histidine metabolism

**Fig. 6:**
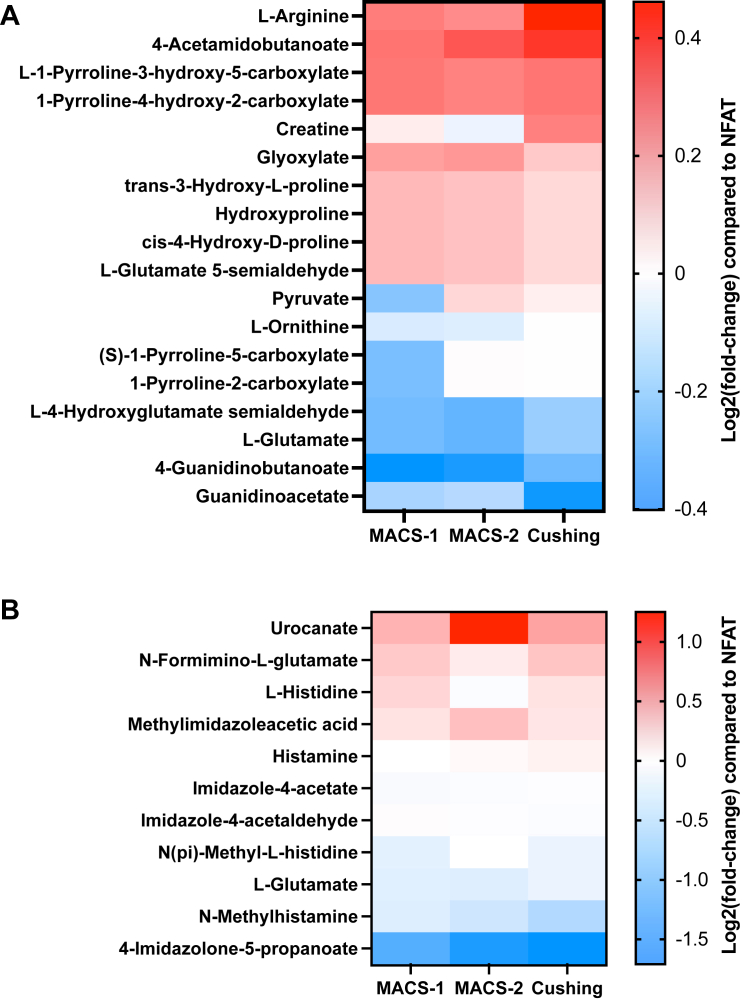
**Pathway enrichment analysis**. Heatmaps of the relative abundances of metabolite features belonging to the arginine and proline metabolism (Panel A) and histidine metabolism (Panel B) pathways, identified by both ordinal regression and generalised matrix learning vector quantisation (see Fig. 4 & “match status” of Supplemental Tables S3 and S4). Results are shown separately for MACS-1 (1 mg-DST cortisol 51–138 nmol/L; n = 70), MACS-2 (1 mg-DST cortisol >138 nmol/L; n = 70), and adrenal Cushing's syndrome (n = 47) as mean fold-changes compared to NFAT (n = 104). Fold-changes are displayed on a log2 scale so that 0 equals no change, and values above and below 0 represent symmetrical degrees of change. Metabolite features are ordered based on abundances in Cushing's syndrome (from highest to lowest). Abbreviations: 1 mg-DST, 1 mg-overnight dexamethasone suppression test; MACS, mild autonomous cortisol secretion; NFAT, non-functioning adrenal tumour.

Pathway enrichment analyses based on metabolite feature ranking by OR (Supplemental Table S3) and GMLVQ (Supplemental Table S4) agreed on identifying the arginine & proline and histidine metabolism as the key pathways affected by cortisol excess (Fig. 4B; Fig. 6; Supplemental Figures S11 and S12). Individuals at higher cardiometabolic risk, i.e., with a diagnosis of hypertension and/or type 2 diabetes, were not robustly identified by the analysis of the untargeted serum metabolome.

### Urinary steroid and serum untargeted metabolome correlation

We identified 210 statistically significant correlations between the five most discriminatory urinary steroid metabolites (Fig. 2B) and 152 serum metabolite features (Supplementary Figures S13 and S14). As expected, the strongest correlations were observed between urinary and serum steroid metabolites. Urinary 11β-hydroxyandrosterone and glucocorticoid metabolites showed the most correlations with lipid metabolites—particularly glycerophospholipids, lysoglycerophospholipids, and ceramides—whereas androsterone primarily correlated with polar metabolites, including those involved in haeme and tryptophan metabolism, and other amino acids (Supplementary Figure S13). Fifteen metabolite features overlapped between 11β-hydroxyandrosterone and tetrahydrocortisone, showing similar magnitude and direction of correlation (Supplementary Figure S15).

## Discussion

This study analysed the steroid and global metabolome in a large prospectively collected cohort of patients with adrenal tumours, EURINE-ACT.^3^^,^^6^ Machine learning showed the expected progressive increase in glucocorticoid excretion and decrease in androgen excretion from NFAT over MACS to CS, which we identified previously.^3^ However, while the classic androgen metabolites decreased, surprisingly, the metabolites of adrenal-derived 11-oxygenated androgens did not. We found that both cortisol excess and increased generation of 11-oxygenated androgens were indicative of higher cardiometabolic risk. Untargeted metabolome analysis revealed a gradual shift towards a lipotoxic phenotype with increasing cortisol excess, which correlated with urinary glucocorticoid and 11-oxygenated androgen metabolite excretion.

Most previous studies looking at the urinary steroid metabolome in patients with benign adrenal tumours were limited by their retrospective nature, and, therefore, more biased patient selection, as well as by much smaller sample sizes. However, small-scale studies utilising gas chromatography-mass spectrometry^11^, ^12^, ^13^, ^14^, ^15^, ^16^, ^17^ and three studies using tandem mass spectrometry^18^, ^19^, ^20^, ^21^ reported some similar findings to our much larger study in prospectively recruited patients, with increased urinary excretion of active glucocorticoid metabolites and increased excretion of THS, the metabolite of the immediate cortisol precursor 11-deoxycortisol. Some of these previous studies also reported decreased androgen metabolite excretion, while, however, analysing only a limited spectrum of androgens. This study reports a differential change in the androgen metabolome, with progressive decreases in the excretion of androsterone and etiocholanolone, the major metabolites of classic androgens testosterone and 5α-dihydrotestosterone, while the adrenal-derived 11-oxygenated androgen metabolite 11β-hydroxyandrosterone did not decrease. Sub-group analysis by sex revealed that women—regardless of the degree of cortisol excess—had overall lower excretion of most glucocorticoid, androgen, and steroid precursor metabolites than men, consistent with known sex differences in adrenal steroidogenesis.^22^

Our study found that patients with adrenal incidentalomas and hypertension exhibited increased glucocorticoid metabolite excretion as compared to those without hypertension; this finding was most pronounced in MACS. Glucocorticoid metabolites were also the most discriminative steroids when differentiating the steroid metabolome of patients with or without hypertension. Though these findings represent no direct evidence of causation, their significance is emphasised by two previous small-scale studies, which reported that serum cortisol in the 1 mg-DST was higher in hypertensive subjects and correlated with waist circumference and the risk of requiring three or more anti-hypertensive medications.^23^^,^^24^ In contrast to our findings in hypertension, we did not observe an obvious correlation between glucocorticoid metabolite excretion and type 2 diabetes, although double GMLVQ subspace learning identified cortisone and the cortisol metabolite 5⍺-THF among the most discriminative steroids for differentiating patients with or without type 2 diabetes.

An intriguing finding of our study is the increased excretion of 11β-hydroxyandrosterone, the major metabolite of adrenal-derived 11-oxygenated androgens, in patients with type 2 diabetes and hypertension, as well as its association with increasing perturbations of the lipidome. The generation of cortisol from 11-deoxycortisol and the 11-oxygenated androgen precursor 11-hydroxyandrostenedione from androstenedione is catalysed by the same enzyme, 11β-hydroxylase (CYP11B1). Increased expression of CYP11B1 has been found in tumour tissue of patients undergoing adrenalectomy for MACS and adrenal CS.^25^^,^^26^ In patients with primary aldosteronism, who regularly exhibit concurrent cortisol excess, the intra-tumoral expression of CYP11B1 has been shown to correlate with the urinary excretion of both cortisol and 11β-hydroxyandrosterone.^14^ The active 11-oxygenated androgen 11-ketotestosterone activates the androgen receptor with equal potency to testosterone,^27^, ^28^, ^29^ and increased androgen bioactivity has been linked to increased risk of cardiometabolic disease in women.^28^ In contrast to gonadal androgens, 11-oxygenated androgens do not decline with age^27^, ^28^, ^29^; therefore, 11-oxygenated androgens represent the dominant androgens in post-menopausal women.

In our study, women constituted most MACS cases (68.7%). Similarly, in a recent retrospective study in a very large pan-European cohort (n = 3565, 42.9% with MACS), 65.7% of patients with MACS were women.^4^ This study reported increased all-cause mortality in women with MACS, but not in men, which was highest in women younger than 65 years^4^; MACS was also linked to increased risk of type 2 diabetes, hypertension and dyslipidaemia. Our findings in the current study show that urinary glucocorticoid metabolites carried the strongest multivariate discriminative information for hypertension and type 2 diabetes in supervised machine learning models (Fig. 3); however, the corresponding effect sizes were modest and heterogeneous across single metabolites (Supplementary Figures S3 and S4). Similarly, 11β-hydroxyandrosterone excretion was modestly increased in patients with benign adrenal tumours and hypertension or type 2 diabetes (Supplementary Figures S3 and S4) and correlated with downstream perturbations of the lipidome (Supplementary Figures S13 and S14). Given that the enzyme CYP11B1 catalyses key steps for both cortisol and 11-oxygenated androgen production, we speculate that there is a link between upregulation of CYP11B1-dependent steroid pathways, higher cortisol and 11-ketotestosterone production, inappropriate prolonged stimulation of the glucocorticoid and androgen receptor in MACS, and cardiometabolic risk.

Studying the untargeted global metabolome in MACS, we found extensive changes in the lipidome and amino acid metabolism, which correlated with the degree of cortisol excess as defined by NFAT, MACS and CS, providing important insights into the lipotoxic metabolic phenotype associated with cortisol excess. Glycerophospholipids were the lipid class most affected in patients with MACS and CS, and perturbations correlated with urinary glucocorticoid metabolite excretion. The changes in glycerophospholipids we observed in our study were primarily characterised by changes in phosphatidylcholines and phosphatidylethanolamines, which are involved in the regulation of lipid metabolism, lipoprotein secretion, and whole-body energy metabolism by supporting mitochondrial function.^30^ Abnormal glycerophospholipid levels in mitochondrial membranes have been linked to the development of mitochondrial dysfunction and cardiometabolic disease.^31^^,^^32^ Phosphatidylcholines and phosphatidylethanolamines can influence insulin signalling in the skeletal muscle, possibly by disrupting calcium signalling,^33^^,^^34^ hence impacting glucose disposal and energy metabolism.^35^^,^^36^ An imbalance of phosphatidylcholines and phosphatidylethanolamines can also lead to dysfunctional membranes of cytosolic lipid droplets, leading to reduced capacity for storing excess lipids in droplets and chronic elevation of circulating fatty acids, resulting in lipotoxicity.^37^^,^^38^ Phosphatidylethanolamines and phosphatidylcholines have been proposed as biomarkers for type 2 diabetes,^39^, ^40^, ^41^ and cardiovascular disease.^39^ Three previous small-scale (n = 20–40) studies in patients with CS^42^, ^43^, ^44^ and MACS^43^ had similar findings, reporting abnormal abundances of several phosphatidylcholines and phosphatidylethanolamines, which appeared negatively associated with cortisol in the 1 mg-DST. Our results in a much larger, prospectively recruited cohort corroborate these findings, demonstrating that the more severe the cortisol excess, the more perturbed the glycerophospholipid profile.

A key finding of our study is the dysregulation of several lipid classes in the lipidome of patients with MACS and CS. Ceramides and other sphingolipids impact mechanisms important for cell cycle regulation, inflammation, angiogenesis, and intracellular trafficking.^45^ An imbalance between these pro-inflammatory lipids and glycerophospholipids has been proposed as a hallmark of lipotoxicity associated with obesity, type 2 diabetes, non-alcoholic fatty liver disease, cardiovascular events, and cancer.^41^^,^^45^^,^^46^ Similarly, lysoglycerophospholipids have been implicated in cell signalling processes underlying immunomodulation, insulin resistance, and endothelial function,^47^ and proposed as biomarkers for obesity, type 2 diabetes, and cardiovascular events.^41^^,^^48^, ^49^, ^50^, ^51^, ^52^

Our comprehensive lipidome analysis identified acylcarnitine perturbations in cortisol excess. Of note, these mitochondrial fatty acid transporters were upregulated in MACS and downregulated in CS. Two previous small-scale studies reported a similar trend towards lower short-/medium-chain acylcarnitines and a relative increase in long-chain acylcarnitines.^42^^,^^43^ Alterations in acylcarnitines point toward dysfunctional lipid β-oxidation and mitochondrial stress, associated with the risk of developing insulin resistance and type 2 diabetes.^41^^,^^53^^,^^54^ A previous study described an acute inhibitory effect of cortisol excess on enzymes involved in lipid β-oxidation.^55^

Pathway enrichment analysis identified abnormal abundances of several amino acids in our patients with MACS and CS, primarily affecting arginine & proline and histidine metabolic pathways. Alterations of arginine and proline metabolism have been linked to type 2 diabetes and cardiovascular disease,^56^, ^57^, ^58^ possibly because of reduced generation of arginine-derived nitric oxide leading to pro-inflammatory changes, mitochondrial dysfunction and oxidative stress.^59^^,^^60^ The disruption of the arginine-nitric oxide pathway has also been observed in experimental models of glucocorticoid-induced hypertension.^61^ Furthermore, arginine is a precursor of polyamines, involved in oxidative stress and cortisol-related immunomodulation^62^; increased levels of the polyamine spermidine have been observed in patients with CS^42^^,^^43^ and linked to the presence of catabolic signs of cortisol excess such as proximal myopathy, skin thinning, and easy bruising.^43^ In our patients with MACS and CS, we observed an overall downregulation of the histidine metabolic pathway (Fig. 6). Histidine has been described to dampen inflammation and ameliorate insulin sensitivity,^63^ and low levels of histidine have been associated with oxidative stress in muscle and sarcopaenia.^64^^,^^65^

Strengths of this study include the large sample size and prospective recruitment, which limits patient selection bias. Untargeted metabolome profiling by mass spectrometry was centralised and with comprehensive coverage of metabolic perturbations using four distinct assays. Untargeted metabolome profiling data are traditionally reported as relative abundance changes between different groups. Whilst this approach provides an overview of the most obvious metabolic perturbations, it does not take into consideration the relationships between different metabolite features, and information that is key for biological interpretation can be lost. Machine learning is a powerful approach to handling these multi-dimensional data and investigating their relationship to outcomes of interest. The reliability of our results is further supported by the close agreement between two machine learning classifiers.

A limitation of our study is its cross-sectional design, which prevents establishing causal relationships between metabolomic alterations and the increased cardiometabolic burden observed in MACS. It remains possible that some of the identified metabolic perturbations are consequences of cardiometabolic comorbidities rather than direct effects of cortisol excess. To minimise this confounding, we performed untargeted metabolome analyses in NFAT subjects matched for BMI and prevalence of hypertension, type 2 diabetes, and dyslipidaemia. Possibly due to the small sample size, we did not observe obvious correlations between untargeted metabolome changes and the presence of cardiometabolic disease. In addition, we included a predominantly female cohort (>80%) to reduce the impact of sex-related differences in the metabolome^66^ and preserve statistical power. This approach is justified by the fact that adrenal incidentalomas disproportionately affect women, and previous studies have shown that women with MACS experience greater adverse effects from cortisol excess, including higher mortality risk.^4^ While our findings are expected to be relevant to both sexes, further studies are warranted to explore potential sex-specific differences. A further limitation is that we did not measure CYP11B1 expression in adrenal tumour tissue, and we could not prove a direct link between increased CYP11B1 activity and overproduction of glucocorticoids and 11-oxygenated androgens; therefore, our results should be interpreted with caution.

In conclusion, cortisol excess was associated with a lipidome signature suggestive of pro-inflammatory changes, incomplete lipid β-oxidation, lipotoxicity, and perturbed amino acid metabolism. Our steroid metabolome and machine learning analyses demonstrated substantial overlap between NFAT and MACS, indicating that these entities are not binary but represent a biological continuum of increasing autonomous cortisol secretion. Recognising this continuum has direct clinical relevance, as it supports a move from rigid diagnostic cut-offs towards risk-based stratification to guide follow-up and treatment decisions. We showed that urinary glucocorticoid and 11-oxygenated androgen metabolites—both downstream of CYP11B1—correlate with hypertension, type 2 diabetes, and lipidome perturbations. This pattern is consistent with a role for CYP11B1-dependent steroidogenesis in cardiometabolic risk. However, tissue validation and interventional studies are required to establish CYP11B1 as a therapeutic target in MACS and to determine whether multi-steroid profiling can refine patient monitoring and inform treatment selection.

## Contributors

A.P. and W.A. designed the study and equally contributed to data collection, analysis, interpretation, supervision, and co-wrote the manuscript. A.P. and W.A. directly accessed and verified the underlying data reported in the manuscript. L.A. and M.C. performed machine learning analyses and co-wrote the manuscript. A.A.Z. and E.L.v.d.B performed machine learning analyses and edited the manuscript. Y.Z. and L.V. contributed to machine learning analyses and edited the manuscript. L.A., M.C., P.T., and M.B. designed the machine learning approaches and developed the double GMLVQ approach. A.J.S., P.T., M.B., and W.B.D. reviewed the statistical and machine learning analyses and edited the manuscript. A.E.T., C.J., and L.C.G. carried out the multi-steroid profiling analysis by mass spectrometry and edited the manuscript. L.N., A.J., G.R.L., and C.L.W. carried out the untargeted metabolome analysis by mass spectrometry, supervised by W.B.D. The co-authors V.C., S.T., K.L., M.M., V.F., I.D.V., G.R., L.V.M., T.D., M.B., M.W.O.R., T.B., T.D., A.D., M.A., A.K., K.Y., J.R.M., M.Q., G.A.U., M.C.D., F.B., A.T., M.F., M.I., M.T., D.K., W.F.Y. Jr, K.N.M., U.A., D.A.V., and I.B. contributed to data collection and edited the manuscript. All authors read and approved the final version of the manuscript.

## Data sharing statement

We have provided a detailed description of the statistical analysis undertaken. We may share de-identified, individual participant-level data that underlie the results reported in this article on receipt of a request detailing the study hypothesis and statistical analysis plan; all requests should be sent to the corresponding author. The analytic codes underpinning the manuscript results are available upon request to be sent to the corresponding author.

## Declaration of interests

A.P., A.E.T., K.L., F.B., and W.A. hold a patent on “Biomarkers for diagnosis and treatment of endocrine hypertension, and methods of identification thereof” (BIO19460); PCT application number: PCT/EP2022/053142. T.B. declares financial relationships with Recordati (speaker honoraria; travel costs).
