# Supplementary material for: Endocrine and metabolic determinants of cardiometabolic risk in mild autonomous cortisol secretion
Source: eBioMedicine. 2026 Jan 22;124:106126. doi: 10.1016/j.ebiom.2026.106126 (PMC12860258; doi:10.1016/j.ebiom.2026.106126)
Supplement: Supplementary material [file mmc1.docx]

**Supplemental material**

[ENSAT EURINE-ACT Investigators 2](#_Toc213858874)

[Methods: Untargeted serum metabolome analysis 4](#_Toc213858875)

[Methods: Supervised machine learning analysis of multi-steroid metabolome data 6](#_Toc213858876)

[Methods: Supervised machine learning analysis of untargeted metabolome data 7](#_Toc213858877)

[Methods: Interpretation of untargeted metabolome data 8](#_Toc213858878)

[Methods: Steroid and untargeted serum metabolome correlation 9](#_Toc213858879)

[Supplemental Table 1 10](#_Toc213858880)

[Supplemental Table 2 11](#_Toc213858881)

[Supplemental Table 3 12](#_Toc213858882)

[Supplemental Table 4 13](#_Toc213858883)

[Supplemental Table 5 14](#_Toc213858884)

[Supplemental Figure 1 15](#_Toc213858885)

[Supplemental Figure 2 16](#_Toc213858886)

[Supplemental Figure 3 17](#_Toc213858887)

[Supplemental Figure 4 18](#_Toc213858888)

[Supplemental Figure 5 19](#_Toc213858889)

[Supplemental Figure 6 20](#_Toc213858890)

[Supplemental Figure 7 21](#_Toc213858891)

[Supplemental Figure 8 22](#_Toc213858892)

[Supplemental Figure 9 23](#_Toc213858893)

[Supplemental Figure 10 24](#_Toc213858894)

[Supplemental Figure 11 25](#_Toc213858895)

[Supplemental Figure 12 26](#_Toc213858896)

[Supplemental Figure 13 27](#_Toc213858897)

[Supplemental Figure 14 28](#_Toc213858898)

[Supplemental Figure 15 29](#_Toc213858899)

[References 30](#_Toc213858900)

# **ENSAT EURINE-ACT Investigators**

List of ENSAT EURINE-ACT Investigators who were involved in the data collection and analysis of the original EURINE-ACT study.^1^ Listed in alphabetical order by country and institution.

^*^ EURINE-ACT investigator who also fulfils authorship criteria for this study (participated in discussion and editorial revision).

Australia

- School of Computing and Information, University of Melbourne, Melbourne, Australia (Stephan Glöckner, Richard O. Sinnott, Anthony Stell)

Brazil

- Adrenal Unit, Division of Endocrinology and Metabolism, Hospital das Clinicas, University of São Paulo Medical School, Institute of Cancer of São Paulo, São Paulo Brazil (Maria Candida B. V. Fragoso)

Croatia

- Department of Endocrinology, University Hospital Centre Zagreb, Zagreb, Croatia (Darko Kastelan^*^, Ivana Dora Vodanovic^*^, Bojana Simunov)

France

- Department of Endocrinology, Hôpital Haut Lévêque, CHU de Bordeaux, Pessac, France (Sarah Cazenave, Magalie Haissaguerre, Antoine Tabarin^*^)
- National Expert Centre for Rare Adrenal Cancers, Cochin Hospital, Institut Cochin, Institut National de la Santé et de la Recherche Medicale Unite 1016, René Descartes University, Paris (Jérôme Bertherat, Rossella Libé)

Germany

- Endocrinology in Charlottenburg, Berlin, Germany (Tina Kienitz, Marcus Quinkler^*^)
- Institute of Clinical Chemistry and Laboratory Medicine, University Hospital Carl Gustav Carus, Technical University, Dresden, Germany (Katharina Langton, Graeme Eisenhofer)
- Medizinische Klinik and Poliklinik IV, Ludwig-Maximilians-Universität München, Munich, Germany (Felix Beuschlein^*^, Christina Brugger, Martin Reincke, Anna Riester, Ariadni Spyroglou)
- Division of Endocrinology and Diabetes, Department of Internal Medicine I, University Hospital, University of Würzburg, German and Comprehensive Cancer Centre Mainfranken, University of Würzburg, Würzburg, Germany (Stephanie Burger-Stritt, Timo Deutschbein^*^, Martin Fassnacht^*^, Stefanie Hahner, Matthias Kroiss, Cristina L. Ronchi)

Greece

- Department of Endocrinology, Diabetes and Metabolism, Evangelismos Hospital, Athens, Greece (Sotiria Palimeri, Stylianos Tsagarakis^*^, Ioanna Tsirou, Dimitra A. Vassiliadi^*^)

Italy

- Department of Clinical and Biological Sciences, San Luigi Hospital, University of Turin, Turin, Italy (Vittoria Basile, Elisa Ingargiola, Giuseppe Reimondo^*^, Massimo Terzolo^*^)
- Department of Experimental and Clinical Biomedical Sciences, University of Florence, Florence, Italy (Letizia Canu, Massimo Mannelli)

The Netherlands

- Department of Internal Medicine, Maxima Medisch Centrum, Eindhoven, The Netherlands (Hester Ettaieb, Harm R. Haak, Thomas M. Kerkhofs)
- Department of Health Services Research, and CAPHRI School for Public Health and Primary Care, Maastricht University, The Netherlands (Harm R. Haak)
- Bernoulli Institute for Mathematics, Computer Science and Artificial Intelligence, University of Groningen, Groningen, The Netherlands (Michael Biehl*)
- Department of Internal Medicine, Division of Endocrinology, Erasmus Medical Centre, University Medical Centre Rotterdam, Rotterdam, The Netherlands (Richard A. Feelders, Johannes Hofland, Leo J. Hofland)

Norway

- Department of Clinical Science, University of Bergen, and Department of Medicine, Haukeland University Hospital, Bergen, Norway (Marianne A. Grytaas, Eystein S. Husebye, Grethe A. Ueland^*^)

Poland

- Department of Internal Medicine and Endocrinology, Medical University of Warsaw, Warsaw, Poland (Urszula Ambroziak^*^, Tomasz Bednarczuk^*^, Agnieszka Kondracka^*^, Magdalena Macech^*^, Malgorzata Zawierucha)

Portugal

- Department of Endocrinology, University Hospital of Coimbra, Coimbra, Portugal (Isabel Paiva)

Republic of Ireland

- School of Medicine, National University of Ireland Galway (NUIG), Galway, Republic of Ireland (M. Conall Dennedy^*^, Ahmed Sajwani)
- Department of Endocrinology, Beaumont Hospital, Dublin, and the Royal College of Surgeons in Ireland, Dublin, Republic of Ireland (Mark Sherlock)
- Department of Endocrinology, St. Vincent’s University Hospital, Dublin, and School of Medicine, University College Dublin, Dublin, Republic of Ireland (Rachel K. Crowley)

Serbia

- Department for Obesity, Reproductive and Metabolic Disorders, Clinic for Endocrinology, Diabetes and Metabolic Diseases, University Clinical Centre of Serbia, Faculty of Medicine, University of Belgrade, Belgrade, Serbia (Miomira Ivovic^*^, Ljiljana V. Marina^*^)

United Kingdom

- Institute of Applied Health Research, University of Birmingham, Birmingham, UK (Jonathan J. Deeks, Alice J. Sitch^*^)
- Institute of Metabolism and Systems Research, University of Birmingham, and Centre for Endocrinology, Diabetes and Metabolism, Birmingham Health Partners, Birmingham, UK (Wiebke Arlt^*^, Irina Bancos^*^, Vasileios Chortis^*^, Lorna C. Gilligan^*^, Beverly A. Hughes, Katharina Lang^*^, Hannah E. Ivison, Carl Jenkinson^*^, Konstantinos Manolopoulos^*^, Donna M. O’Neil, Michael W. O‘Reilly^*^, Thomas G. Papathomas, Alessandro Prete^*^, Cristina L. Ronchi, Cedric H.L. Shackleton, Angela E. Taylor^*^)
- Department of Endocrinology, Queen Elizabeth Hospital, University Hospitals Birmingham NHS Foundation Trust, Birmingham, UK (Wiebke Arlt^*^, Miriam Asia^*^, Vasileios Chortis^*^, Katharina Lang^*^, Konstantinos N. Manolopoulos^*^, Michael W. O’Reilly^*^, Alessandro Prete^*^, Cristina L. Ronchi)
- Department of Hepato-Pancreato-Biliary and Liver Transplant Surgery, Queen Elizabeth Hospital, University Hospitals Birmingham NHS Foundation Trust, Birmingham, UK (Robert P. Sutcliffe)
- Department of Radiology, Queen Elizabeth Hospital, University Hospitals Birmingham NHS Foundation Trust, Birmingham, UK (Peter Guest)
- Department of Pathology, Queen Elizabeth Hospital, University Hospitals Birmingham NHS Foundation Trust, Birmingham, UK (Kassiani Skordilis)

United States of America

- Division of Endocrinology, Metabolism and Nutrition, Mayo Clinic, Rochester, MN, USA (Irina Bancos^*^, Cristian Bancos, Alice Chang, Caroline J. Davidge-Pitts, Danae A. Delivanis, Dana Erickson, Neena Natt, Todd B. Nippoldt, Melinda Thomas, William F. Young Jr.^*^)
- UCSF Benioff Children’s Hospital Oakland Research Institute, Oakland, California, CA, USA (Cedric H.L. Shackleton)

# **Methods: Untargeted serum metabolome analysis**

The samples were aliquoted on the day of collection and stored locally at -80 °C. The samples were transported on dry ice to the University of Birmingham, United Kingdom, and stored at -80 °C before untargeted serum metabolome profiling.

Sample extraction

***Serum samples:*** Samples were randomised using the RAND() function in Microsoft Excel to determine the sample preparation order. This order was assessed to ensure that all the metadata (age, sex, body mass index, and disease classification) was randomised and there were no patterns/correlation with the sample preparation order. Serum samples were thawed on ice on the day of preparation. For the hydrophilic interaction chromatography (HILIC) assay, serum (50 μL) and 1/1 acetonitrile/methanol (v/v) (150 μL) were mixed by vortex (120 s) and centrifuged (17,000 *g*, 20 min, 4°C). For lipid assays, serum (50 μL) and isopropanol (150 μL) were mixed by vortex (120 s) and centrifuged (17,000 g, 20 min, 4°C).

***Pooled quality control (QC) samples:*** 120 µL serum aliquots from all biological samples were transferred to a single tube and mixed by vortex. This single-tube sample is called the pooled QC sample. Multiple 50 μL aliquots were prepared as described for serum samples.

***Process blank samples:*** Blank extraction solutions were prepared by performing the extraction protocol as described above for samples with no serum present.

Ultra-high performance liquid chromatography-mass spectrometry (UHPLC-MS)

All three types of samples were analysed by applying two UHPLC-MS methods in positive and negative ion modes separately using a Dionex UltiMate 3000 UHPLC system coupled with a heated electrospray Q Exactive Focus mass spectrometer (Thermo Fisher Scientific). The analysis order for the samples was randomised, and all the samples were analysed in two analytical batches. Ten QC samples were injected at the start of each assay/ion mode to condition the analytical system and were then injected after every 6th biological sample, with two QC samples being analysed at the end of each batch. The blank sample was analysed as the 5th and as the final injection of each batch.

***HILIC assays:*** These assays used an Accucore150-Amide-HILIC column (100 x 2·1mm, 2·6μm, Thermo Fisher Scientific). For positive ion analysis, mobile phase A was 10 mM ammonium formate dissolved in acetonitrile/water/formic acid (95:4·9:0·1 (v/v)) and mobile phase B was 10 mM ammonium formate dissolved in acetonitrile/water/formic acid (50/49·9/0·1 (v/v)). For negative ion analysis, mobile phase A was 10 mM ammonium acetate dissolved in acetonitrile/water/acetic acid (95:4·9:0·1 (v/v)) and mobile phase B was 10 mM ammonium acetate dissolved in acetonitrile/water/acetic acid (50/49.9/0.1 (v/v)). The gradient elution applied for positive and negative ion mode was t=0·0, 1% B; t=1·0, 1% B; t=3·0, 15% B; t=6·0, 50% B; t=9·0, 95% B; t=10·0, 95% B; t=10·5, 1% B; t=14·0, 1% B. All changes were linear (curve=5) and the flow rate was 0·50 mL.min-1. The column temperature was 35°C and the injection volume was 2 μL. Data were acquired in positive and negative ionisation modes separately (70 – 1050 *m/z*) with a mass resolution of 70,000 (FWHM, *m/z* 200). Ion source parameters applied were Sheath gas = 55 arbitrary units, Aux gas = 35 arbitrary units, Sweep gas = 4 arbitrary units, Spray Voltage = 3·2kV (positive ion) and 2·7kV (negative ion), Capillary temp. = 380°C, Aux gas heater temp. = 440°C. Thermo Exactive Tune (2·8 SP1, build 2806) software controlled the instruments and data acquisition. All data were collected as MS1 data in profile mode with the exception of five QC sample injections (injections 6-10) where MS/MS data were collected in the “Discovery mode” setting over different precursor *m/z* ranges (70-210 *m/z*; 200-310 *m/z*; 300-410 *m/z*; 400-510 *m/z*; 500-1050 *m/z*) using stepped normalized collision energies (positive ion mode: 20, 40, 100%; negative ion mode: 40, 60, 130%). All samples were maintained at a temperature of 4°C in the autosampler. Instrument maintenance (source and column cleaning) was performed between the end of batch 1 and the start of batch 2.

***Lipid assays:*** These assays used a reversed-phase Hypersil GOLD C_18_ column (100 x 2·1 mm, 1·9μm; Thermo Fisher Scientific). Mobile phase A was 10 mM ammonium formate dissolved in acetonitrile/water/formic acid (60:39·9:0·1 (v/v)) and mobile phase B was 10 mM ammonium formate dissolved in isopropanol/acetonitrile/water/formic acid (85·5/9·5/4·9/0·1 (v/v)). The gradient elution applied was t=0·0, 20% B; t=0·5, 20% B, t=8·5, 100% B; t=9·5, 100% B; t=11·5, 20% B; t=14·0, 20% B. All changes were linear (curve=5) and the flow rate was 0·40 mL/min. Column temperature was 55 °C and injection volume was 2 μL. Data were acquired in positive and negative ionisation modes separately (150-2000 *m/z*) with a mass resolution of 70,000 (FWHM, *m/z* 200). Ion source parameters applied were Sheath gas = 48 arbitrary units, Aux gas = 15 arbitrary units, Sweep gas = 0 arbitrary units, Spray Voltage = 3·2kV (positive ion) / 2·7kV (negative ion), Capillary temp. = 380°C, Aux gas heater temp. = 450°C. Thermo Exactive Tune (2·8 SP1, build 2806) software controlled the instruments and data acquisition. All data were acquired in profile mode. All data were collected as MS1 data in profile mode with the exception of five QC sample injections (injections 6-10) where MS/MS data were collected in the “Discovery mode” setting over different precursor m/z ranges (150-510 *m/z*; 500-710 *m/z*; 700-860 *m/z*; 850-1010 *m/z*; 1000-2000 *m/z*) using stepped normalised collision energies (positive ion mode: 20, 40, negative ion mode: 40, 60, 130%). All samples were maintained at a temperature of 4°C in the autosampler. Instrument maintenance (source and column cleaning) was performed between the end of batch 1 and the start of batch 2.

***Raw data processing:*** Vendor format raw data files (.RAW) were converted to the mzML file format using the open-source ProteoWizard software.^2^ Deconvolution was performed by the R package XCMS (version 3.6.1 running in the Galaxy environment).^3^ The R package IPO^4^ was used to optimise and obtain XCMS peak-picking parameters for each of the different assays:

(a) HILIC negative ion mode assay: min peak width = 6·23; max peak width = 40; ppm = 9·98; mzdiff = -0·02025; bw = 0·25; mzwid = 0·001; minfrac = 0·5; snr_thr = 10.

(b) HILIC positive ion mode assay: min peak width = 5·70; max peak width = 40; ppm = 7·0; mzdiff = 0·00021; bw = 0·25; mzwid = 0·0059; minfrac = 0·5; snr_thr = 10.

(c) Lipids negative and positive ion mode assays: min peak width = 5·44; max peak width = 40; ppm = 6·5; mzdiff = -0·0065; bw = 0·25; mzwid = 0·04265; minfrac = 0·5; snr_thr = 10.

A data matrix of metabolite features (i.e. *m/z*-retention time pairs) versus samples was constructed for each of four assay types (HILIC-negative, HILIC-positive, Lipids-negative, Lipids-positive).

***Data quality assessment and filtering:*** Each of the four data matrices constructed by raw data processing was filtered based on the data collected for QC and blank samples.^5^ Metabolite features were retained in the data matrix if they were: present in >90% of all the QC samples, had a peak area relative standard deviation (RSD) <30% across all the QC samples (QC11 onwards) and had an extract blank/mean QC area ratio of <5%.

***Metabolite annotation:*** Metabolite annotation was performed applying the Python package BEAMSpy^6^ (RT diff = 2 s, Pearson correlation >0·50; *m/z* values of all experimentally observed peaks were searched against LIPIDMAPS^7^ and HMDB,^8^ all matches < 5ppm mass error tolerance were reported). To generate more robust compound annotations using MS/MS data, QC sample UHPLC-MS/MS data were matched to MS/MS databases using the LipidSearch software (lipid assay metabolite annotation; version 4.2.18, Thermo Fisher Scientific) and were searched against the entire *in-silico* HCD MS/MS database with 5ppm mass error. HILIC MS/MS data were searched in mzCloud using Compound Discoverer (v3.1, Thermo Fisher Scientific) using a >70% match as the criteria for a successful annotation. RT data were matched to an in-house RT library constructed with authentic chemical standards, and matches within 5 seconds were applied as the criteria for a successful match. LipidSearch and mzCloud annotations were aligned to the XCMS outputs using the R programming language (<https://www.R-project.org>), applying a 5ppm mass error for the MS1 data and a 5s retention time tolerance window. All annotations described are reported to either level 2 or 3 as defined by the Metabolomics Standards Initiative.^9^

***Pathway enrichment analysis:*** Pathway enrichment analysis was performed in the software MetaboAnalyst^10^ for HILIC-based results. Pathway enrichment analysis applied pathway analysis, scatter plot for visualization method, hypergeometric test for enrichment method, relative-betweenness centrality for topology analysis and Homo sapiens (KEGG) as the pathway library. Only pathways with a p-value (after FDR correction) < 0·05 and with a minimum of 5 metabolites in the pathway listed as biologically important were reported. For lipid-based results, lipids were classified according to LipidMaps ontologies^7^ manually.

# **Methods: Supervised machine learning analysis of multi-steroid metabolome data**

Generalised matrix learning vector quantisation (GMLVQ): The mathematical details of GMLVQ, a prototype-based classification method and extension of Learning Vector Quantisation (LVQ) have been described elsewhere.^11-14^ GMLVQ has been applied to multi-steroid urinary metabolome data in previous studies.^1,15,16^ In brief, 24-hour excretion values of the steroid metabolites were log-transformed and subsequently z-score normalised to the means and standard deviations observed in the dataset. The resulting set of multi-dimensional vectors (one dimension for each steroid), together with the class membership (non-functioning adrenal tumours [NFAT], possible mild autonomous cortisol secretion [MACS-1], definitive MACS [MACS-2], adrenal Cushing’s syndrome [CS], hypertension yes/no, type 2 diabetes yes/no, bilateral tumours yes/no) served as input for the machine learning analysis. For each classifier experiment, the dataset was randomly split into a training and a validation set (90% and 10% of cases, respectively), which were used to evaluate the performance of GMLVQ in each experiment. We used a stratified train-test split to ensure that class distributions were preserved across training and testing sets. Each experiment was repeated at least 30 times, and the performance was measured as the average area under the receiver-operating characteristic curve (AUC-ROC). In each run, the data set was balanced independently to the smallest class by undersampling the majority-classes ^17^. We consider the adrenal CS class as the minority class, as it has the least number of samples. All other classes are considered as the majority classes. Since we have different numbers of samples in each class, we under-sampled the NFAT, MACS-1, and MACS-2 classes to have the same number of samples as the adrenal CS class. The relevance of steroid metabolites for the classification was determined as the indices of the highest values of the relevance matrix diagonal on average over the multiple training processes ^18^. In this analysis, the GMLVQ implementation Version 3.0 from ^14^ was used.

Ordinal regression (OR): OR refers to a setting in which data items belong to categories/classes organised in a linear order. As principally classification problems, OR problems can be addressed by regular classification techniques; however, algorithms which explicitly account for the class order are preferred because they naturally penalise less for misclassifications of classes close in the order than for those at the order extremes. The extension of LVQ to OR algorithms^19-22^ and multi-class classification^23^ are examples of OR. Unlike the previous OR extensions of LVQ methods, the Accumulative Ordinal Generalised Matrix Learning Vector Quantisation (a-OGMLVQ) ^19^ considers a unified cost for all prototypes, and the bandwidth of the prototypes’ weights is adapted automatically. If the class distribution in the data space in an OR problem indeed reflects the expected class order, the additional model constraint in dedicated OR classifiers imposing the class order can be beneficial, resulting in a more robust positioning of the learnt class prototypes. However, if this is not the case, the less constrained GMLVQ models can be more appropriate. Even though we naturally expect that our data features do organise the ordered classes in a spatially ordered fashion, we nevertheless, besides the OR classifiers also train the unconstrained GMLVQ models as informative baselines.

Discriminative subspace emersion to identify the most relevant features of clinical outcomes in benign adrenal tumours according to the degree of cortisol excess: A methodology to recover the subspace of most relevant steroid metabolomes was used to discriminate between patients with and without clinical characteristics of interest (hypertension, type 2 diabetes, bilateral tumours) between two groups: MACS and NFAT. The theoretical framework of this method is described in^24^ and addresses the following question: *What are the steroid metabolites that have consistently different importance across two populations (NFAT or MACS) when distinguishing between patients with or without a certain clinical outcome (e.g. hypertension)?* Briefly, the subspace learning framework was extended into a general approach for identifying faint signals in stratified classification. It is shown how the features diverging most in relevance tend to emerge even when the classification performance itself is poor in the individual populations. The method involves a double application of a subspace learning algorithm (e.g., GMLVQ, Support Vector Machine) on the multi-steroid metabolome data of two populations (MACS and NFAT) labelled according to binary clinical characteristics (hypertension yes/no, type 2 diabetes yes/no, bilateral tumours yes/no). In the first phase, the method identifies and ranks relevant steroid features of binary clinical characteristics individually in MACS and NFAT. In the second phase, it distinguishes between already learned subspaces to determine which steroid metabolite groups differ the most in distinguishing clinical characteristics between MACS and NFAT. Even if the performance in the first phase is suboptimal due to highly overlapping urinary steroid excretion, discriminative subspace emersion can identify the separation direction quickly and effectively. This new methodology allows performing a multivariate analysis of the data and provides an in-depth evaluation of the relevance of the steroid features, overcoming the noise deriving from highly overlapping datasets having more than one set of labels.

# **Methods: Supervised machine learning analysis of untargeted metabolome data**

Data modelling and interpretation involved the following steps:

1. Between 2500 and 3000 metabolite features were identified and quantified (varying numbers based on the UHPLC-MS method and ion mode used). The relative abundances were expressed as normalised peak areas (mean ± 95%CI).
2. The relative abundances of the metabolite features were log-transformed to reduce the skewness of the dataset and then normalised (z-score).
3. The high dimensionality of the data set was reduced via principal component analysis (PCA). By computing the cumulative variance explained by the eigenvectors of the covariance matrix (ordered in decreasing order of their corresponding eigenvalues), the ones cumulative explaining 80% of the variance were retained. This led to reducing the dimensionality of the HILIC and lipid data sets down to 60 principal components (eigenvectors). Given the linear nature of PCA, the projection matrix onto the lower-dimensional space is invertible and its inverse is used after the classification (step 5) to recover the importance (relevance) of the original metabolite features.
4. Missing data were handled by probabilistic PCA (PPCA).^25^ This method constructs a generative probabilistic model of the complete data, i.e., the observed data and the missing values. In particular, the model is constructed as a “pancake-shaped” multivariate Gaussian with the principal axis of variation organised along a low-dimensional linear latent space optimally embedded in the higher-dimensional data space, convolved with a spherical Gaussian noise. The missing observations are also treated as latent variables. The model is trained in the Maximum Likelihood Estimation (MLE) framework through an Expectation-Maximisation (EM) algorithm.^26^ The trained PPCA generative model is then used to produce multiple imputed data sets, where for each data item with missing observations, the missing values are imputed by sampling from the posterior distribution provided by the PPCA, given the observed features for that data item. The main advantage over other imputation techniques (e.g. k-nearest neighbours) is the global, potentially more robust, nature of the multivariate imputation model. In our untargeted metabolome dataset, data were missing at random across all four assays used (Supplemental Table 5).
5. Metabolomic data were analysed using prototype-based supervised machine learning: GMLVQ and OR. GMLVQ and OR were used to determine the relevance of each metabolite feature for the classification of benign adrenal tumours across the spectrum of autonomous cortisol secretion, i.e., the four-class classification NFAT – MACS-1 – MACS-2 – adrenal CS. The relevance of each metabolite feature was expressed as median rank and interquartile range (the smaller the rank, the more relevant that metabolite feature). Due to the imputation procedure with PPCA, given a dataset with missing values, 30 alternative datasets were created as explained above – these datasets preserved the observed features but imputed the missing values by sampling from the PPCA model distribution, conditioned on the observed data. Fifty individual classifiers (either OR or GMLVQ) were trained on the 50 alternative datasets and then combined into a single ensemble classifier (with flat weighting) using a majority vote to produce the final classification decision. The final feature ranks were obtained from the ensemble classifiers by averaging all the relevance matrices obtained in the reduced space spanned by the dominant principal components, found with PCA. The average relevance matrix was then de-projected back into the original metabolite feature space by inverting the projection matrix from step 3. Metabolite features were then ranked by their position in the recovered feature relevance list.
6. Since two UHPLC-MS methods were used (HILIC and C_18_; each using two ion modes) and two classifiers applied, eight lists of ordered metabolite features were generated (four for GMLVQ and four for OR) (**Supplemental Figure 15**).
7. After visual inspection of the plots of the metabolite features ordered by their relevance, an arbitrary cut-off of the top 500 metabolites was chosen for biological interpretation (**Supplemental Figure 15**). This cut-off was beyond the knee point of the ranked features and was deemed to offer an acceptable compromise between including too much noise (i.e., the risk of including metabolite features that are not very relevant for the classification of autonomous cortisol secretion and may mask underlying metabolic processes) or too little information (i.e., the risk of excluding metabolite features that point towards key metabolic perturbations linked to autonomous cortisol secretion).
8. The four ranking lists generated by GMLVQ were merged and the same was done for OR, yielding one list of metabolite features for GMLVQ and one list of metabolite features for OR. Duplicate metabolite features across the various ranking lists were removed.

# **Methods: Interpretation of untargeted metabolome data**

The metabolite feature abundances in patients with MACS-1, MACS-2, and adrenal CS were expressed as fold-changes using patients with NFAT as the reference group. This analysis was carried out separately for the metabolite lists generated by GMLVQ and OR. Moreover, non-polar and polar metabolite features were analysed separately.

Non-polar metabolite features (measured by C_18_ reversed-phase UHPLC-MS): The lipidome was analysed by grouping non-polar metabolite features into classes related to chemical structure or metabolic function. Only classes where 20 or more metabolite features were selected by GMLVQ or OR were brought forward for biological interpretation. This arbitrary cut-off was deemed sufficient to identify biologically meaningful changes in patients with MACS and adrenal CS whilst reducing the risk of reporting spurious results linked to the inherent variability of the metabolome.

Polar metabolites (measured by HILIC UHPLC-MS): Pathway enrichment analysis was used to identify which metabolic pathways had metabolite features that were over-represented and had significant perturbations to their abundances. One-way ANOVA with *post hoc* analysis to correct for multiple comparisons using false discovery rate was used to identify the most affected metabolic pathways in patients with MACS and adrenal CS; p-values <0·05 were considered statistically significant. Pathway enrichment analysis was performed using MetaboAnalyst,^10^ and box and whisker plots were constructed using data normalised by sum.^27^

# **Methods: Steroid and untargeted serum metabolome correlation**

Correlations between absolute urinary steroid concentrations and normalised relative abundances of serum metabolite features were assessed using Spearman rank analysis in MetaboAnalyst.^10^ Correlation analyses were limited to the top 500 ranked serum metabolite features identified by supervised machine learning (GMLVQ and OR analysed separately) and the top 5 ranked steroid metabolites (cortisol, androsterone, tetrahydrocortisone, pregnenetriol, and 11β-hydroxyandrosterone) identified by OR across the spectrum of increasing cortisol excess (NFAT, MACS-1, MACS-2, and Cushing’s syndrome; **Figure 2B**). Given the exploratory nature of this analysis, a conservative p-value threshold of 0·0005 was applied to identify statistically significant correlations for biological interpretation.

# **Supplemental Table 1**

**24-hour urinary steroid excretion measured by liquid chromatography-tandem mass spectrometry (LC-MS/MS).**

Steroid excretion is expressed in µg/24h and reported as median (interquartile range) in 24-h urine collected by patients with non-functioning adrenal tumours (NFAT), mild autonomous cortisol secretion (MACS-1 and MACS-2 listed separately), and clinically overt Cushing’s syndrome (CS). There were no missing data in the urinary steroid metabolome.

| **Metabolite** | **NFAT (n=649)** | **MACS-1 (n=451)** | **MACS-2 (n=140)** | **Adrenal CS (n=65)** |
| --- | --- | --- | --- | --- |
| **Core steroid precursor metabolites** | | | | |
| Pregnenediol (5-PD) | 81 (55-144) | 64 (55-106) | 56 (55-105) | 89 (55-158) |
| Pregnanediol (PD) | 328 (190-597) | 281 (157-479) | 254 (149-503) | 536 (206-813) |
| Pregnenetriol (5-PT) | 92 (49-177) | 63 (43-126) | 56 (43-101) | 71 (43-115) |
| Pregnanetriol (PT) | 333 (179-567) | 257 (143-465) | 210 (118-452) | 222 (145-368) |
| 17α-Hydroxypregnanolone (17HP) | 69 (39-135) | 63 (37-127) | 51 (32-108) | 74 (45-116) |
| Tetrahydro-11-deoxycortisol (THS) | 141 (87-222) | 142 (91-239) | 177 (90-271) | 317 (181-500) |
| **Glucocorticoid metabolites** | | | | |
| Cortisol (F) | 45 (28-65) | 54 (32-82) | 57 (33-92) | 151 (76-344) |
| Tetrahydrocortisol (THF) | 1362 (914-2011) | 1460 (888-2165) | 1563 (998-2293) | 3163 (1466-6425) |
| 5α-Tetrahydrocortisol (5α-THF) | 568 (287-986) | 543 (267-947) | 506 (206-823) | 642 (315-1088) |
| 11β-Hydroxyetiocholanolone (11β-OHEt) | 305 (120-541) | 335 (135-613) | 413 (156-769) | 602 (182-1310) |
| Cortisone (E) | 73 (47-105) | 76 (47-108) | 82 (49-115) | 141 (95-317) |
| Tetrahydrocortisone (THE) | 2223 (1457-3409) | 2181 (1334-3329) | 2323 (1296-3170) | 3812 (1939-5865) |
| β-Cortolone | 634 (401-964) | 624 (389-989) | 658 (341-937) | 998 (622-1632) |
| **Androgen and androgen precursor metabolites** | | | | |
| Androsterone (An) | 577 (258-1034) | 290 (127-642) | 191 (97-474) | 167 (61-314) |
| Etiocholanolone (Et) | 540 (264-1073) | 364 (167-747) | 329 (144-689) | 331 (221-725) |
| Dehydroepiandrosterone (DHEA) | 26 (22-54) | 22 (22-30) | 22 (22-24) | 22 (22-22) |
| 11β-Hydroxyandrosterone (11β-OHAn) | 541 (292-874) | 551 (306-973) | 523 (254-778) | 524 (295-847) |

# **Supplemental Table 2**

**Most relevant non-polar metabolite features for the classification of benign adrenal tumours based on machine learning analysis.**

The number of metabolite features identified by generalised matrix learning vector quantisation (GMLVQ), ordinal regression (OR), as well as the number of overlapping features between the two classifiers, are listed separately. Only metabolite classes were classifiers identified ≥20 features were selected for further analysis (shown in bold).

| **Metabolite class** | **Number of metabolite features identified by machine learning** | | |
| --- | --- | --- | --- |
|  | **GMLVQ** | **OR** | **overlapping** |
| **Glycerophospholipids (GPLs)** | 297 | 207 | 82 |
| GPLs (phosphatidylcholine, PC) | 82 | 49 | 19 |
| GPLs (phosphatidylinositol, PI) | 22 | 23 | 10 |
| GPLs (phosphatidylethanolamine, PE) | 26 | 16 | 4 |
| GPLs (phosphatidylserine, PS) | 15 | 12 | 1 |
| GPLs (phosphatidic acid, PA) | 11 | 8 | 3 |
| GPLs (phosphatidylglycerol, PG) | 9 | 5 | 1 |
| GPLs (mixed)* | 132 | 94 | 44 |
| **Lysoglycerophospholipids** | 76 | 80 | 35 |
| **Triacylglycerides** | 70 | 72 | 33 |
| **Ceramides** | 65 | 73 | 24 |
| **Sphingolipids** | 53 | 42 | 23 |
| **Acylcarnitines** | 22 | 21 | 12 |
| Oxidised fatty acids | 11 | 7 | 4 |
| Fatty acids | 9 | 13 | 4 |
| Bile acids | 7 | 4 | 3 |
| Cardiolipins | 7 | 8 | 2 |
| Diacylglycerides | 6 | 9 | 6 |
| Acyl amino acids | 4 | 6 | 1 |
| Cholesterol metabolism | 8 | 6 | 4 |
| Steroid metabolism | 3 | 1 | 1 |
| GPL synthesis | 3 | 3 | 1 |
| Vitamin A metabolism | 2 | 3 | / |
| Vitamin D metabolism | 1 | / | / |
| Mixed class^†^ | 39 | 37 | 17 |
| Peptides^†^ | 22 | 16 | 6 |
| * This includes metabolite features that belong to the glycerophospholipid class, but it is unknown which subclass they belong to. Therefore, they have been included in the overall analysis of the glycerophospholipid class but have been excluded from subclass analyses.  ^†^ These classes include metabolite features that have not been attributed to specific metabolite classes. Therefore, they have not been used for biological interpretation. | | | |

# **Supplemental Table 3**

**Pathway enrichment analysis based on metabolite feature ranking by ordinal regression.**

Pathway enrichment analysis was carried out on the top 500 polar metabolite features identified by hydrophilic interaction chromatography (HILIC) ultra-high performance liquid chromatography-mass spectrometry (UHPLC-MS) in the positive and negative ion modes and ranked in order of relevance by ordinal regression (OR). The “match status” column shows the number of metabolites belonging to a specific pathway that OR assigned to the top 500 over the total number of metabolites in that pathway (e.g., 17 out of 38 metabolites of the arginine and proline metabolism pathway are found in the top 500 list generated by OR). The “annotated compound names” column reports the list of metabolites belonging to a specific pathway; the metabolites found in the top 500 list generated by OR (match status) are shown in bold. Abbreviations: FDR, false discovery rate.

| **Pathway Name** | **FDR-corrected p-value** | **Match Status** | **Annotated compound names** |
| --- | --- | --- | --- |
| Arginine and proline metabolism | <0·0001 | 17/38 | **L-Arginine**; N(omega)-Hydroxyarginine; **Guanidinoacetate**; **Creatine**; 4-Aminobutanoate; Agmatine; Putrescine; 4-Aminobutyraldehyde; S-Adenosylmethioninamine; S-Adenosyl-L-methionine; Spermidine; N-Acetylputrescine; N4-Acetylaminobutanal; Spermine; **trans-3-Hydroxy-L-proline**; D-Proline; (4R)-4-Hydroxy-2-oxoglutarate; L-erythro-4-Hydroxyglutamate; **L-4-Hydroxyglutamate semialdehyde**; **L-1-Pyrroline-3-hydroxy-5-carboxylate**; **Hydroxyproline**; L-Proline; **cis-4-Hydroxy-D-proline**; **(S)-1-Pyrroline-5-carboxylate**; L-Glutamyl 5-phosphate; **L-Glutamate**; **L-Glutamate 5-semialdehyde**; **L-Ornithine**; Peptide; Nitric oxide; Phosphocreatine; 4-Guanidinobutanoate; **4-Acetamidobutanoate**; Homocarnosine; **1-Pyrroline-2-carboxylate**; **Glyoxylate**; **Pyruvate**; **1-Pyrroline-4-hydroxy-2-carboxylate** |
| Histidine metabolism | <0·0001 | 10/16 | **L-Glutamate**; **4-Imidazolone-5-propanoate**; Urocanate; **L-Histidine**; Carnosine; **Imidazole-4-acetaldehyde**; **N-Methylhistamine**; Methylimidazole acetaldehyde; **Histamine**; **N(pi)-Methyl-L-histidine**; N-Formyl-L-aspartate; **N-Formimino-L-glutamate**; beta-Alanyl-N(pi)-methyl-L-histidine; **Imidazole-4-acetate**; **Methylimidazoleacetic acid**; L-Aspartate |
| Valine, leucine, and isoleucine biosynthesis | 0·0029 | 6/8 | **L-Threonine**; **(S)-3-Methyl-2-oxopentanoic acid**; **L-Leucine**; 3-Methyl-2-oxobutanoic acid; **2-Oxobutanoate**; **L-Isoleucine**; 4-Methyl-2-oxopentanoate; **L-Valine** |
| Alanine, aspartate, and glutamate metabolism | 0·0309 | 10/28 | **N-Acetyl-L-aspartate**; 2-Oxosuccinamate; L-Aspartate; **L-Asparagine**; D-Aspartate; N-(L-Arginino)succinate; N6-(1,2-Dicarboxyethyl)-AMP; L-Alanine; **Succinate semialdehyde**; **L-Glutamate**; 4-Aminobutanoate; **L-Glutamine**; Ammonia; **2-Oxoglutaramate**; **(S)-1-Pyrroline-5-carboxylate**; N-Acetylaspartylglutamate; N-Acetylaspartylglutamylglutamate; Citrate; Oxaloacetate; Fumarate; **Pyruvate**; **N-Carbamoyl-L-aspartate**; **Succinate**; 2-Oxoglutarate; Carbamoyl phosphate; D-Glucosamine 6-phosphate; 5-Phosphoribosylamine; beta-Citryl-L-glutamate |

# **Supplemental Table 4**

**Pathway enrichment analysis based on metabolite feature ranking by generalised matrix learning vector quantisation.**

Pathway enrichment analysis was carried out on the top 500 polar metabolite features identified by hydrophilic interaction chromatography (HILIC) ultra-high performance liquid chromatography-mass spectrometry (UHPLC-MS) in the positive and negative ion modes and ranked in order of relevance by generalised matrix learning vector quantisation (GMLVQ). The “match status” column shows the number of metabolites belonging to a specific pathway that GMLVQ assigned to the top 500 over the total number of metabolites in that pathway (e.g., 12 out of 38 metabolites of the arginine and proline metabolism pathway are found in the top 500 list generated by GMLVQ). The “annotated compound names” column reports the list of metabolites belonging to a specific pathway; the metabolites found in the top 500 list generated by GMLVQ (match status) are shown in bold. Abbreviations: FDR, false discovery rate.

| **Pathway Name** | **FDR-corrected p-value** | **Match status** | **Annotated compound names** |
| --- | --- | --- | --- |
| Arginine and proline metabolism | 0·0015 | 12/38 | **L-Arginine**; N(omega)-Hydroxyarginine; Guanidinoacetate; **Creatine**; 4-Aminobutanoate; Agmatine; Putrescine; 4-Amino-butyraldehyde; S-Adenosylmethioninamine; S-Adenosyl-L-methionine; Spermidine; N-Acetylputrescine; N4-Acetylamino-butanal; Spermine; **trans-3-Hydroxy-L-proline**; D-Proline; (4R)-4-Hydroxy-2-oxoglutarate; L-erythro-4-Hydroxyglutamate; **L-4-Hydroxyglutamate semialdehyde**; **L-1-Pyrroline-3-hydroxy-5-carboxylate**; **Hydroxyproline**; L-Proline; **cis-4-Hydroxy-D-proline**; (S)-1-Pyrroline-5-carboxylate; L-Glutamyl 5-phosphate; L-Glutamate; **L-Glutamate 5-semialdehyde**; **L-Ornithine**; Peptide; Nitric oxide; Phosphocreatine; **4-Guanidinobutanoate**; 4-Acetamidobutanoate; Homocarnosine; 1-Pyrroline-2-carboxylate; Glyoxylate; **Pyruvate**; **1-Pyrroline-4-hydroxy-2-carboxylate** |
| Histidine metabolism | 0·0401 | 6/16 | L-Glutamate; **4-Imidazolone-5-propanoate**; **Urocanate**; L-Histidine; Carnosine; Imidazole-4-acetaldehyde; **N-Methylhistamine**; Methylimidazole acetaldehyde; Histamine; **N(pi)-Methyl-L-histidine**; N-Formyl-L-aspartate; **N-Formimino-L-glutamate**; beta-Alanyl-N(pi)-methyl-L-histidine; Imidazole-4-acetate; **Methylimidazoleacetic acid**; L-Aspartate |

# **Supplemental Table 5**

**Missing values in the untargeted metabolome dataset.**

Data were missing at random across all four assays used. We report the percentage of missing values per column across the untargeted metabolome dataset, expressed as minimum, maximum, median, mode, and interquartile range (IQR). Abbreviations: HILIC, hydrophilic interaction chromatography.

| **Assay** | **Minimum (%)** | **Maximum (%)** | **Median (%)** | **Mode (%)** | **IQR (%)** |
| --- | --- | --- | --- | --- | --- |
| HILIC – negative ion | 2·5 | 28·4 | 5·8 | 5·2 | 2·6 |
| HILIC – positive ion | 1·8 | 19·7 | 4·4 | 4·4 | 2·5 |
| C18 reversed-phase – negative ion | 1·0 | 21·0 | 3·5 | 3·1 | 2·0 |
| C18 reversed-phase – positive ion | 1·1 | 12·3 | 3·1 | 2·3 | 1·9 |

# **Supplemental Figure 1**

**Urinary steroid excretion across benign adrenocortical tumours with different degrees of autonomous cortisol secretion.**

The 24-hour urinary excretion of each steroid metabolite in patients with MACS-1 (1mg-DST cortisol 51-138 nmol/L), MACS-2 (1mg-DST cortisol >138 nmol/L), and adrenal Cushing’s syndrome is compared to patients with NFAT by using linear regression models adjusted for age, sex, and body mass index. Percentage changes are reported as sympercents. Abbreviations: 1mg-DST, 1mg-overnight dexamethasone suppression test; MACS, mild autonomous cortisol secretion; NFAT, non-functioning adrenal tumour. For steroid abbreviations, see Supplemental Table 1. Data derived from.^28^

# **Supplemental Figure 2**

**Urinary steroid excretion stratified by sex and age across benign adrenocortical tumours with different degrees of autonomous cortisol secretion.**

The 24-hour urinary excretion of each steroid metabolite in patients with NFAT (1mg-DST cortisol ≤50 nmol/L), MACS-1 (1mg-DST cortisol 51-138 nmol/L), MACS-2 (1mg-DST cortisol >138 nmol/L), and adrenal Cushing’s syndrome is compared to the reference group by using linear regression models adjusted for age, sex, and body mass index. Percentage changes are reported as sympercents. Panel A: Women vs. men (reference). Panel B: Patients aged >60 years versus ≤60 years (reference; median age of the entire cohort = 60 years). Abbreviations: 1mg-DST, 1mg-overnight dexamethasone suppression test; MACS, mild autonomous cortisol secretion; NFAT, non-functioning adrenal tumour. For steroid abbreviations, see Supplemental Table 1.

# **Supplemental Figure 3**

**24-hour urinary steroid excretion in patients with non-functioning adrenal tumours (NFAT) and mild autonomous cortisol secretion (MACS), with and without hypertension.**

The urinary excretion of each metabolite in patients with hypertension was compared to patients without hypertension (reference) by using a linear regression model with the log-transformed steroid metabolite as the outcome (adjusted for age, sex, and body mass index). Associations between the log-transformed outcome and the variable of interest are reported as sympercents (mean percentage change and 95% confidence interval). Patients with MACS were analysed together with NFAT (left panels) and separately (right panels).

# **Supplemental Figure 4**

**24-hour urinary steroid excretion in patients with non-functioning adrenal tumours (NFAT) and mild autonomous cortisol secretion (MACS), with and without type 2 diabetes.**

The urinary excretion of each metabolite in patients with type 2 diabetes was compared to patients without type 2 diabetes (reference) by using a linear regression model with the log-transformed steroid metabolite as the outcome (adjusted for age, sex, and body mass index). Associations between the log-transformed outcome and the variable of interest are reported as sympercents (mean percentage change and 95% confidence interval). Patients with MACS were analysed together with NFAT (left panels) and separately (right panels).

# **Supplemental Figure 5**

**24-hour urinary steroid excretion in patients with non-functioning adrenal tumours (NFAT) and mild autonomous cortisol secretion (MACS), with and without bilateral adrenal masses.**

The urinary excretion of each metabolite in patients with bilateral adrenal masses was compared to patients with unilateral masses (reference) by using a linear regression model with the log-transformed steroid metabolite as the outcome (adjusted for age, sex, and body mass index). Associations between the log-transformed outcome and the variable of interest are reported as sympercents (mean percentage change and 95% confidence interval). Patients with MACS were analysed together with NFAT (left panels) and separately (right panels).

# **Supplemental Figure 6**

**Lipidome perturbations observed by generalised matrix learning vector quantisation in patients with benign adrenal tumours and cortisol excess.**

Relative abundances of metabolite features belonging to the six lipid classes identified by generalised matrix learning vector quantisation. Results are shown separately for MACS-1 (1mg-DST cortisol 51-138 nmol/L), MACS-2 (1mg-DST cortisol >138 nmol/L), and adrenal CS as fold-changes compared to NFAT. Fold-changes are displayed on a log2 scale so that 0 equals no change, and values above and below 0 represent symmetrical degrees of change. Results are shown as boxplots, with boxes representing the median and interquartile range, and whiskers representing the 5th to 95th centile. Results are shown separately for all features combined, increased features only, and decreased features only. Abbreviations: 1mg-DST, 1mg-overnight dexamethasone suppression test; CS, Cushing’s syndrome; MACS, mild autonomous cortisol secretion; NFAT, non-functioning adrenal tumour.

# **Supplemental Figure 7**

**Perturbations of selected lipid metabolite features.**

Relative abundances of the nine annotated lipid metabolites with the highest statistical significance based on a one-way ANOVA statistical analysis (p<0·05, FDR-corrected). Results are shown separately for NFAT (1mg-DST cortisol ≤50 nmol/L), MACS-1 (1mg-DST cortisol 51-138 nmol/L), MACS-2 (1mg-DST cortisol >138 nmol/L), and adrenal CS. Results are shown as boxplots, with boxes representing the median and interquartile range, and whiskers representing the 5th to 95th centile. Abbreviations: 1mg-DST, 1mg-overnight dexamethasone suppression test; Cer, ceramide; CS, Cushing’s syndrome; FDR, false discovery rate; HexCer, hexoseceramide; LPE, lysophosphatidylethanolamine; MACS, mild autonomous cortisol secretion; NFAT, non-functioning adrenal tumour; PC, phosphatidylcholine; PE, phosphatidylethanolamine; PI, phosphatidylinositol; TG, triacylglyceride.

# **Supplemental Figure 8**

**Glycerophospholipid subclass perturbations observed by ordinal regression in patients with benign adrenal tumours and cortisol excess.**

Relative abundances of metabolite features belonging to six glycerophospholipid subclasses identified by ordinal regression. Results are shown separately for MACS-1 (1mg-DST cortisol 51-138 nmol/L), MACS-2 (1mg-DST cortisol >138 nmol/L), and adrenal CS as fold-changes compared to NFAT. Fold-changes are displayed on a log2 scale so that 0 equals no change, and values above and below 0 represent symmetrical degrees of change. Results are shown as boxplots, with boxes representing the median and interquartile range, and whiskers representing the 5th to 95th centile. Results are shown separately for all features combined, increased features only, and decreased features only. Abbreviations: 1mg-DST, 1mg-overnight dexamethasone suppression test; CS, Cushing’s syndrome; MACS, mild autonomous cortisol secretion; NFAT, non-functioning adrenal tumour; PA, phosphatidic acids; PC, phosphatidylcholines; PE, phosphatidylethanolamines; PI, phosphatidylinositols; PG, phosphatidylglycerols; PS, phosphatidylserines.

# **Supplemental Figure 9**

**Glycerophospholipid subclass perturbations observed by generalised matrix learning vector quantisation in patients with benign adrenal tumours and cortisol excess.**

Relative abundances of metabolite features belonging to six glycerophospholipid subclasses identified by generalised matrix learning vector quantisation. Results are shown separately for MACS-1 (1mg-DST cortisol 51-138 nmol/L), MACS-2 (1mg-DST cortisol >138 nmol/L), and adrenal CS as fold-changes compared to NFAT. Fold-changes are displayed on a log2 scale so that 0 equals no change, and values above and below 0 represent symmetrical degrees of change. Results are shown as boxplots, with boxes representing the median and interquartile range, and whiskers representing the 5th to 95th centile. Results are shown separately for all features combined, increased features only, and decreased features only. Abbreviations: 1mg-DST, 1mg-overnight dexamethasone suppression test; CS, Cushing’s syndrome; MACS, mild autonomous cortisol secretion; NFAT, non-functioning adrenal tumour; PA, phosphatidic acids; PC, phosphatidylcholines; PE, phosphatidylethanolamines; PI, phosphatidylinositols; PG, phosphatidylglycerols; PS, phosphatidylserines.

# **Supplemental Figure 10**

**Acylcarnitine perturbations observed by supervised machine learning in patients with benign adrenal tumours and cortisol excess.**

Relative abundances of acylcarnitines identified by supervised machine learning. Results are shown separately for MACS-1 (1mg-DST cortisol 51-138 nmol/L), MACS-2 (1mg-DST cortisol >138 nmol/L), and adrenal Cushing’s syndrome as fold-changes compared to NFAT. Fold-changes are displayed on a log2 scale so that 0 equals no change, and values above and below 0 represent symmetrical degrees of change. The Y-axis corresponds to the rank of each acylcarnitine according to supervised machine learning (the lower the rank, the more relevant the metabolite feature is for adrenal tumours associated with cortisol excess). Acylcarnitines are color-coded by class: short-chain (C_2_ to C_5_, black); medium-chain (C_6_ to C_12_, light blue); long-chain (C_13_ to C_18_, green); very long-chain (C_19+_, red). Abbreviations: 1mg-DST, 1mg-overnight dexamethasone suppression test; MACS, mild autonomous cortisol secretion; NFAT, non-functioning adrenal tumour.

# **Supplemental Figure 11**

**Perturbations observed in the arginine and proline metabolism pathway.**

The metabolites identified by supervised machine learning are circled in red. Enzymes active in humans are shown in green. Generated with KEGG ^29^.

# **Supplemental Figure 12**

**Perturbations observed in the histidine metabolism pathway.**

The metabolites identified by supervised machine learning are circled in red. Generated with KEGG ^29^.

# **Supplemental Figure 13**

**Overview of the urinary steroid and serum untargeted metabolome correlation.**

Number of metabolite features among the top 500 that significantly correlated with the five steroid metabolites discriminating non-functioning adrenal tumours, mild autonomous cortisol secretion, and adrenal Cushing’s syndrome (Figure 2B). Correlations were assessed by Spearman rank analysis, and associations with p < 0·0005 were considered statistically significant. Results are shown separately for polar (right panel) and non-polar metabolites (left panel); the circle areas are directly proportional to the number of features. Annotated lipid classes and polar metabolites are described, some features were not matched to a metabolite or class

# **Supplemental Figure 14**

**Urinary steroid and serum untargeted metabolome correlation coefficients.**

Heatmap showing the statistically significant correlation coefficients (Spearman rank analysis, p <0·0005) between cortisol (F), androsterone (An), tetrahydrocortisone (THE), pregnenetriol (5-PT), 11β-hydroxyandrosterone (11β-OHAn), and 152 metabolite features among the top 500 ranked by supervised machine learning. Non-annotated metabolite features are numbered (#). Blank cells indicate non-significant correlations. Abbreviations: AC, acylcarnitine; Cer, ceramide; CholMet, cholesterol metabolism; CL, cardiolipin; DG, diacylglycerides; FA, fatty acids; GPL, glycerophospholipid; LGPL, lysoglycerophospholipid; OxFa, oxidised fatty acids; StMet, steroid metabolism; TG, triacylglycerides; VitDMet, vitamin D metabolism.

# **Supplemental Figure 15**

**Selection of untargeted metabolome features by supervised machine learning.**

Four datasets were generated (HILIC UHPLC-MS, negative ion mode; HILIC UHPLC-MS, positive ion mode; C_18_ reversed-phase UHPLC-MS, negative ion mode; C_18_ reversed-phase UHPLC-MS, positive ion mode) and the metabolite features ranked by generalised matrix learning vector quantisation (GMLVQ) and ordinal regression (OR). The lower the rank, the more relevant a metabolite feature is for the classifications of persons with mild autonomous cortisol secretion and adrenal Cushing’s syndrome. The red dotted lines show the chosen cut-off of 500 metabolite features. Abbreviations: HILIC, hydrophilic interaction chromatography; UHPLC-MS, ultra-high performance liquid chromatography-mass spectrometry.

# **References**

1. Bancos I, Taylor AE, Chortis V, et al. Urine steroid metabolomics for the differential diagnosis of adrenal incidentalomas in the EURINE-ACT study: a prospective test validation study. *Lancet Diabetes Endocrinol* 2020; **8**(9): 773-81.

2. Chambers MC, Maclean B, Burke R, et al. A cross-platform toolkit for mass spectrometry and proteomics. *Nat Biotechnol* 2012; **30**(10): 918-20.

3. Smith CA, Want EJ, O'Maille G, Abagyan R, Siuzdak G. XCMS: processing mass spectrometry data for metabolite profiling using nonlinear peak alignment, matching, and identification. *Anal Chem* 2006; **78**(3): 779-87.

4. Libiseller G, Dvorzak M, Kleb U, et al. IPO: a tool for automated optimization of XCMS parameters. *BMC Bioinformatics* 2015; **16**: 118.

5. Lloyd GR, Jankevics A, Weber RJM. struct: an R/Bioconductor-based framework for standardized metabolomics data analysis and beyond. *Bioinformatics* 2021; **36**(22-23): 5551-2.

6. <https://github.com/computational-metabolomics/beamspy>

7. Conroy MJ, Andrews RM, Andrews S, et al. LIPID MAPS: update to databases and tools for the lipidomics community. *Nucleic Acids Res* 2024; **52**(D1): D1677-D82.

8. Wishart DS, Guo A, Oler E, et al. HMDB 5.0: the Human Metabolome Database for 2022. *Nucleic Acids Res* 2022; **50**(D1): D622-D31.

9. Sumner LW, Amberg A, Barrett D, et al. Proposed minimum reporting standards for chemical analysis Chemical Analysis Working Group (CAWG) Metabolomics Standards Initiative (MSI). *Metabolomics* 2007; **3**(3): 211-21.

10. Pang Z, Lu Y, Zhou G, et al. MetaboAnalyst 6.0: towards a unified platform for metabolomics data processing, analysis and interpretation. *Nucleic Acids Res* 2024; **52**(W1): W398-W406.

11. Schneider P., Biehl M., Hammer B. Adaptive relevance matrices in learning vector quantization. Neural Comput. 2009;21(12):3532-61.

12. Biehl M, Schneider P, Smith D, Stiekema H, Taylor AE, Hughes B, et al. Matrix relevance LVQ in steroid metabolomics based classification of adrenal tumors. In: ESANN. 2012.

13. Biehl M, Hammer B, Villmann T. Prototype-based models in machine learning. In: Wiley Interdisciplinary Reviews: Cognitive Science 7.2 (2016), pp. 92–111.

14. Biehl M. A collection of no-nonsense GMLVQ demo code. 2020. url: <https://www.cs.rug.nl/~biehl/gmlvq>.

15. Arlt W, Biehl M, Taylor AE, et al. Urine steroid metabolomics as a biomarker tool for detecting malignancy in adrenal tumors. *J Clin Endocrinol Metab* 2011; **96**(12): 3775-84.

16. Prete A, Lang K, Pavlov D, et al. Urine steroid metabolomics as a diagnostic tool in primary aldosteronism. *J Steroid Biochem Mol Biol* 2024; **237**: 106445.

17. Kubat M. An introduction to machine learning. Springer, 2017.

18. Schneider P, Biehl M, Hammer B. Adaptive relevance matrices in learning vector quantization. *Neural Comput* 2009; **21**(12): 3532-61.

19. Tang F., Tiño P. Ordinal regression based on learning vector quantization, Neural Networks 93 (2017) 76–88, doi: 10.1016/j.neunet.2017.05.006.

20. Fouad S., Tiño P. Adaptive Metric Learning Vector Quantization for Ordinal Classification, Neural computation 24 11 (2012) 2825–2851.

21. Chu W., Keerthi SS. Support Vector Ordinal Regression, Neural Comput. 19 (3) (2007-03) 792–815, doi: 10.1162/neco.2007.19.3.792.

22. Shashua A., Levin A. Ranking with Large Margin Principle: Two Approaches, in: Proceedings of the 15th International Conference on Neural Information Processing Systems, in: NIPS’02, MIT Press, 2002, pp. 961–968. <http://dl.acm>. org/citation.cfm?id=296 8618.296 8738.

23. Frank E., Hall M. A Simple Approach to Ordinal Classification, in: L. De Raedt, P. Flach (Eds.), Machine Learning: ECML 2001, 2001, pp. 145–156.

24. <https://arxiv.org/abs/2504.00176>

25. Tipping MEB, C. M. Probabilistic Principal Component Analysis. *Journal of the Royal Statistical Society Series B: Statistical Methodology* 1999; **61**(3): 611–22.

26. Rubin DBT, D. T. EM algorithms for ML factor analysis. *Psychometrika* 1982; **47**(1): 69–76.

27. Chong J, Wishart DS, Xia J. Using MetaboAnalyst 4.0 for Comprehensive and Integrative Metabolomics Data Analysis. *Curr Protoc Bioinformatics* 2019; **68**(1): e86.

28. Prete A, Subramanian A, Bancos I, et al. Cardiometabolic Disease Burden and Steroid Excretion in Benign Adrenal Tumors : A Cross-Sectional Multicenter Study. *Ann Intern Med* 2022; **175**(3): 325-34.

29. Kanehisa M. Toward understanding the origin and evolution of cellular organisms. *Protein Sci* 2019; **28**(11): 1947-51.
